# Supplementary material for: VARP Is Recruited on to Endosomes by Direct Interaction with Retromer, Where Together They Function in Export to the Cell Surface
Source: Dev Cell. 2014 Jun 9;29(5):591–606. doi: 10.1016/j.devcel.2014.04.010 (PMC4059916; doi:10.1016/j.devcel.2014.04.010)
Supplement: Document S1. Article plus Supplemental Experimental Procedures, Figures S1–S6, and Tables S1 and S2 [file mmc1.pdf]

Developmental Cell, Volume *29*

Supplemental Information

VARP Is Recruited onto Endosomes by Direct Interaction with Retromer, Where Together They Function in Export to the Cell Surface

Geoffrey G. Hesketh, Inmaculada Pérez-Dorado, Lauren P. Jackson, Lena Wartosch, Ingmar B. Schäfer, Sally R. Gray, Airlie J. McCoy, Oliver B. Zeldin, Elspeth F. Garman, Michael E. Harbour, Philip R. Evans, Matthew N.J. Seaman, J. Paul Luzio, and David J. Owen

Figure S1. Structure of the VARP ANKRD1:Rab32 complex
Related to Figure 1

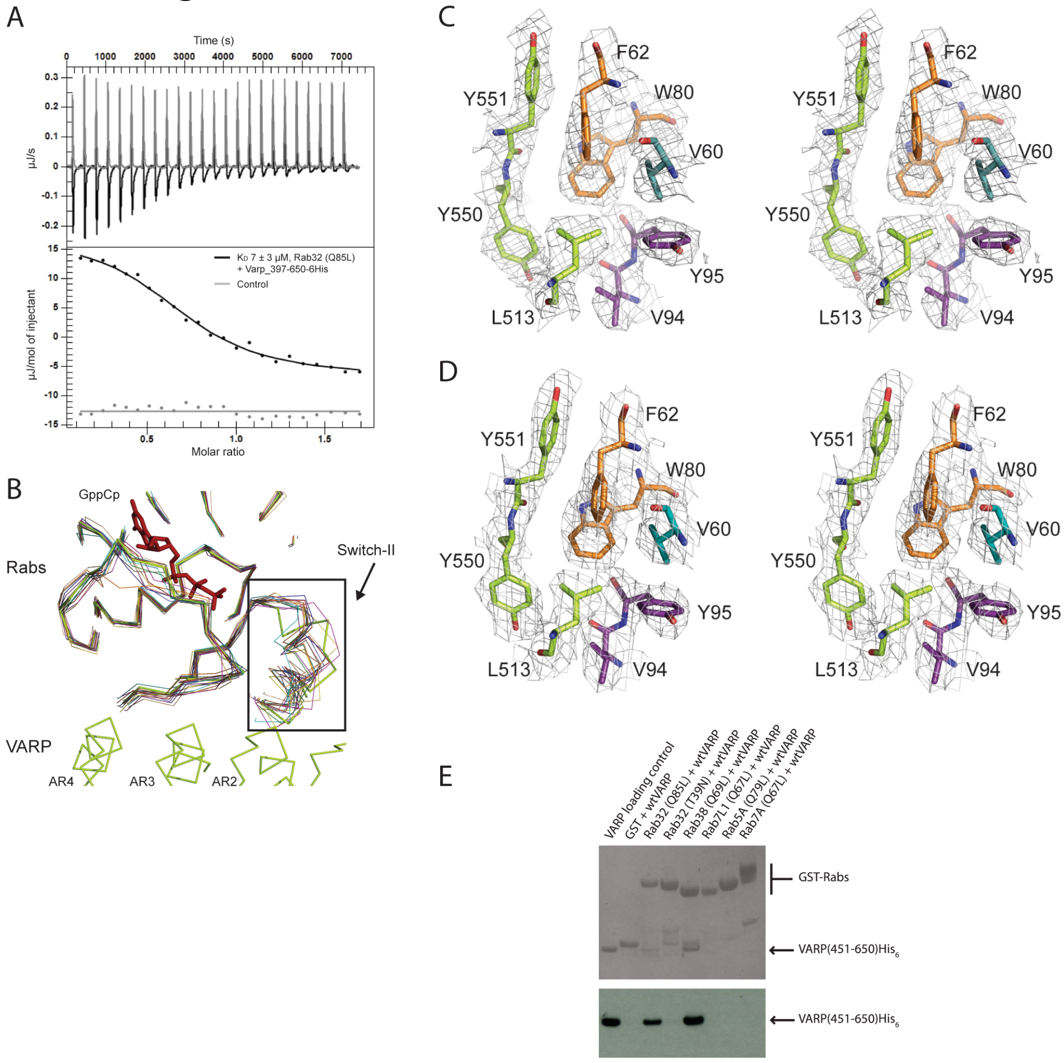

FIGURE S1

(A) Binding of VARP residues 397-650 to Rab32(Q85L) residues 1-225 analysed by ITC gave an estimated K_D of 7±3µM.

(B) Differences in the conformation of the GTPase in VARP ANKRD1:Rab32 complex compared to those in other Rab:effector complexes. Ribbon representation VARP:Rab32 (thicker and in green) superimposed with other Rab:GTPs: 1ZBD (yellow); 1TUE (red); 1Z0J (pink); 1Z0k (orange); 2D7C (light yellow); 2HV8 (magenta); 3BBP (purple); 2ZET (grey); 1YHN (cyan); 3BC1 (green-cyan); 3MJH (light blue); 3QBT (dark blue); 3TNF (brown); 3TKL (dark-green); and 3CWZ (beige). The Switch II region is highlighted by boxing. The only Rab effector shown is VARP.

(C) and (D), stereoviews showing a detail of the final refined 2mFo-DFc electron density maps contoured at 1.0 sigma for native (C) and SeMet-derivative VARP-(D) ANKRD1:Rab32 complexes.

(E) 'GST pull downs' using VARP451-640His$_6$ and GST tagged versions of the GTP locked forms of the endosomal Rabs, Rab5, Rab7, Rab7L1, Rab32 and Rab38 to show the specificity of the interaction for Rab32/38.

Figure S2 Analysis of the 'cross dimer' of the VARP-ANKRD1:Rab32 heterodimer.
Related to Figure 2

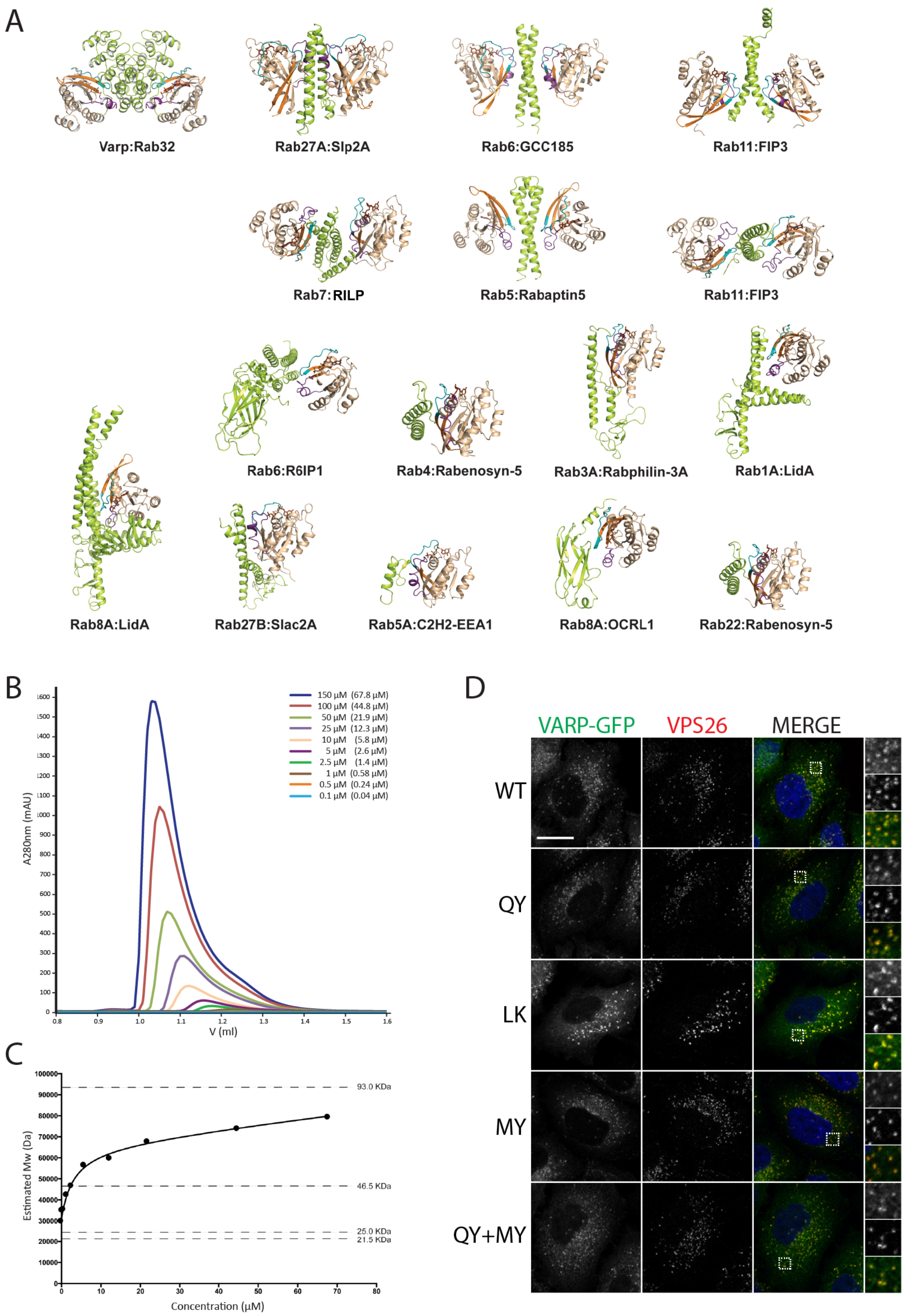

FIGURE S2

(A) Structures of various Rab:Rab effector complexes. VARP:Rab32 (4CYM); Rab27A:Slp2A (3BC1); Rab6:GCC185 (3BBP); Rab11:FIP3 (2HV8); Rab7:RILP (1YHN); Rab5:Rabaptin5 (1TUE); Rab11:FIP3 (2D7C); Rab6:R6IP1 (3CWZ);

Rab3A:Rabphilin-3A (1ZBD); Rab1A:LidA (3TKL); Rab8A:LidA (3TNF); Rab27B:Slac2A (2ZET); Rab5A:C2H2-EEA1 (3MJH); Rab8:OCRL1 (3QBT); Rab4:Rabenosyn-5 (1Z0K); and Rab22:Rabenosyn-5 (1Z0J). The color code throughout is green for the Rab-effectors, and beige for the Rabs with Switch-I, inter-switch, and Switch-II regions highlighted in cyan, orange, and purple respectively.

(B) and (C) Analytical gel filtration of VARP ANKRD1:Rab32(Q85L) complex. Samples of the complex were loaded at ten different concentrations ranging from 0.1 μM to 150 μM. In each case, calculated concentrations in the peak are indicated in brackets. Superimposition of the ten chromatograms (B) shows a concentration dependent shift of the peak to higher molecular weight as sample concentration is increased. Values of estimated molecular weight and protein concentrations at the peak were fitted to a saturation-binding curve (C). The molecular weight estimated at saturation is consistent with the molecular weight of the heterotetramer (93 KDa). Values of the molecular weight for the monomeric proteins VARP (21.5 KDa) and Rab32 (25 KDa), heterodimer (46.5 KDa) and heterotetramer (93 KDa) are indicated.

(D) IF confocal microscopy of VARP-GFP-HeLa cells without cytosol extraction (relating to Figure 2H) (GFP (green), VPS26 (red), nuclei (blue)) showing that disrupting the VAMP7 and/or Rab32 interaction with VARP did not inhibit the recuitment of VARP onto endosomes. Boxed regions in the merged panels are shown as separate green (top), red (middle) and merged (bottom) channels on the right. Scale bar = 20μm

Figure S3. VARP is recruited to endosomes by retromer.
Related to Figure 3

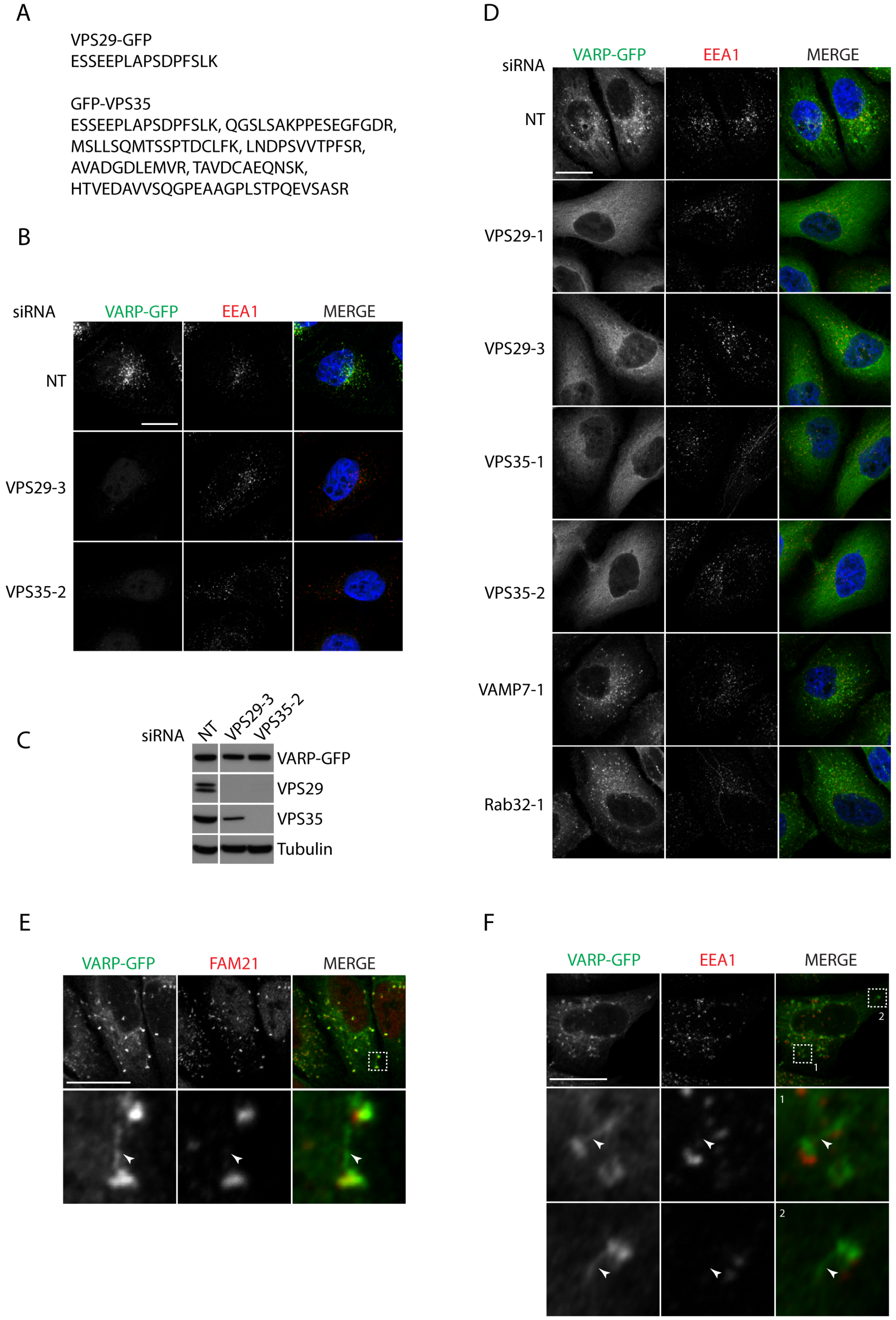

FIGURE S3

(A) VARP peptides identified by anti-GFP native co-immunoprecipitation and mass spectrometry analysis using HeLa cells stably expressing VPS29-GFP or GFP-VPS35. No VARP peptides were detected in a similar control lysate of untransfected HeLa cells treated with anti-GFP.
(B) IF confocal microscopy of cytosol extracted VARP-GFP-HeLa cells knocked-down with single siRNA oligonucleotides at 100nM (NT = non-targeting control, VPS29-3, VPS35-2), (GFP (green), EEA1 (red), nuclei (blue, merged panels)).
(C) Western blots of the cells imaged in B showing successful protein depletion. The VPS29-3 and VPS35-2 images are from cropped regions of the same Western blots that appear in Figure 3B, and the NT images are identical to those in Figure 3B.
(D) IF confocal microscopy of VARP-GFP-HeLa cells without cytosol extraction relating to Figure 3A and S3B (GFP (green), EEA1 (red), nuclei (blue, merged panels)).
(E)(F) IF confocal microscopy of VARP-GFP-HeLa cells without cytosol extraction (GFP (green), FAM21 (red)(E) or EEA1 (red)(F)). The boxed regions are shown in the bottom row with the VARP-GFP positive tubular domains of endosomes indicated with arrowheads. Scale bars = 20μm

Figure S4. VARP binds to a conserved hydrophobic patch on VPS29, which also binds to TBC1D5.
Related to Figure 4

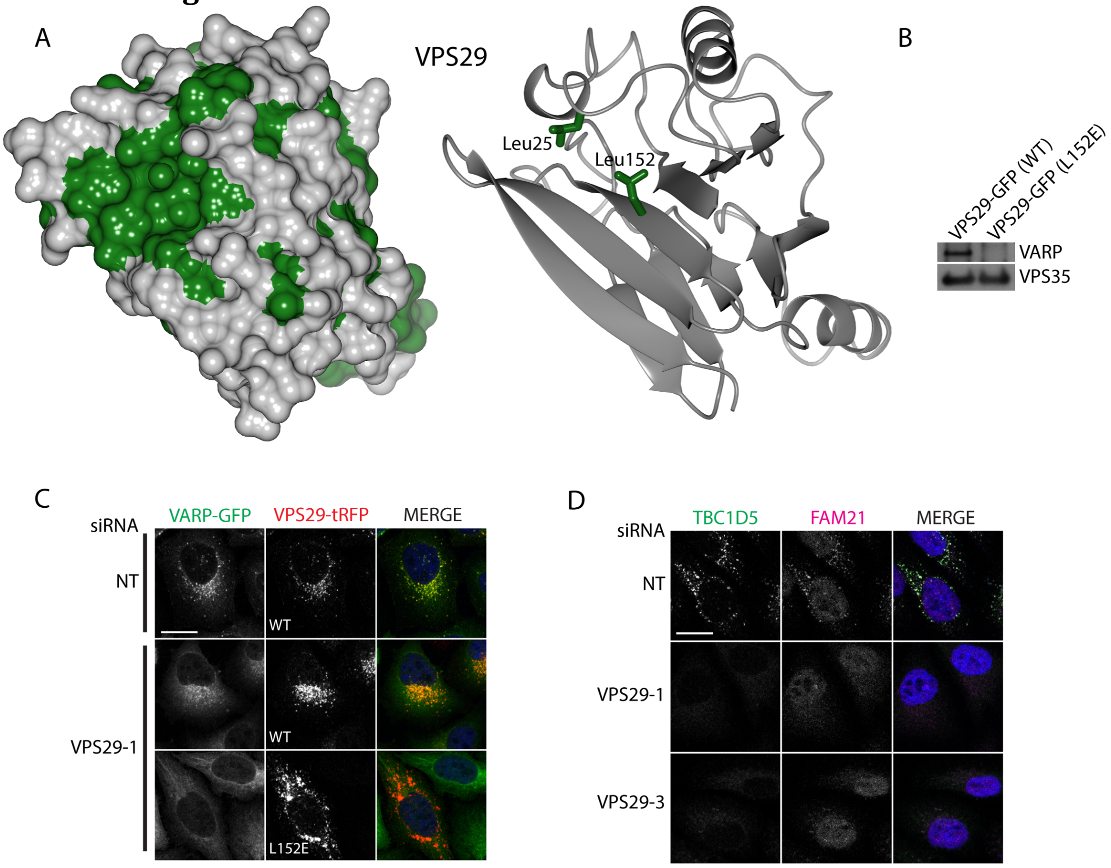

FIGURE S4

(A) Structure of VPS29 in surface representation (PDB code 1Z2W) with exposed hydrophobic side chains coloured in green (left panel) and in ribbon representation with Leu25 and Leu152, mutation of which abolishes both VARP and TBC1D5 binding, indicated (right panel).

(B) Anti-GFP native co-immunoprecipitation of HeLa cells expressing wild-type (WT) or L152E mutant VPS29-GFP, Western blotted for VARP and VPS35.

(C) IF confocal microscopy of VARP-GFP-HeLa cells without cytosol extraction relating to Figure 4D.

(D) IF confocal microscopy of cytosol extracted VARP-GFP-HeLa cells knocked-down using single siRNA oligonucleotides at 100nM (NT = non-targeting control, VPS29-1, VPS29-3) (TBC1D5 (green) and FAM21 (magenta), nuclei (blue, merged panels)). Scale bars = 20µm

Figure S5. VARP binds to VPS29 via two conserved Zn^{2+} coordinating Cys-rich motifs.
Related to Figure 5

A

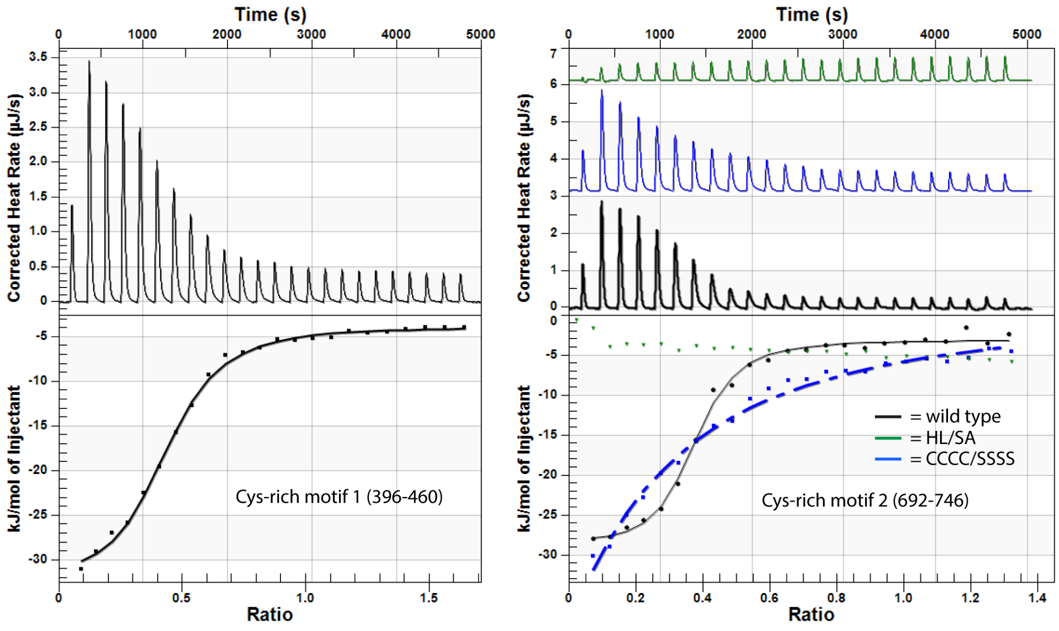

B

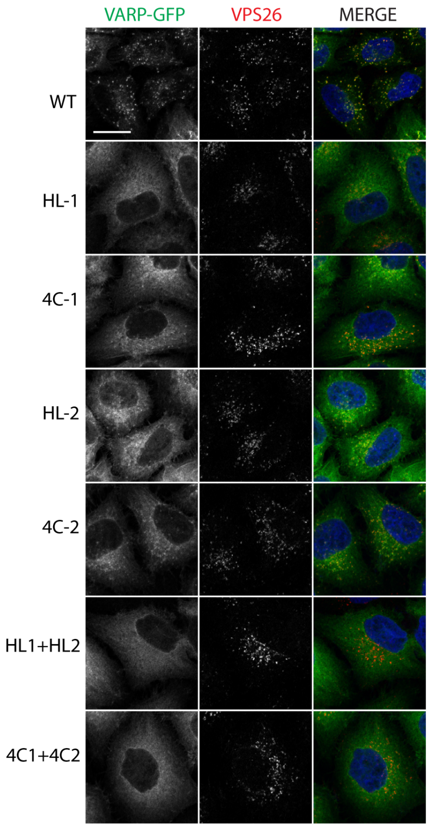

FIGURE S5

(A) Isothermal titration calorimetry (ITC) traces of VARP Cys-rich motif 1 (residues 396-460) and motif 2 (residues 692-746) binding to VPS29. For motif 2, H712S/L714A (HL/SA) (green trace) and C711S/C715S/C717S/C720S (CCCC/SSSS) (blue trace) mutations were tested.

(B) IF confocal microscopy of HeLa cells expressing wild type (WT) or mutated VARP-GFP, without cytosol extraction, related to Figure 5E. Scale bar = 20μm.

Figure S6. The VARP/retromer/VAMP7 protein network is required for GLUT1 trafficking.
Related to Figure 6

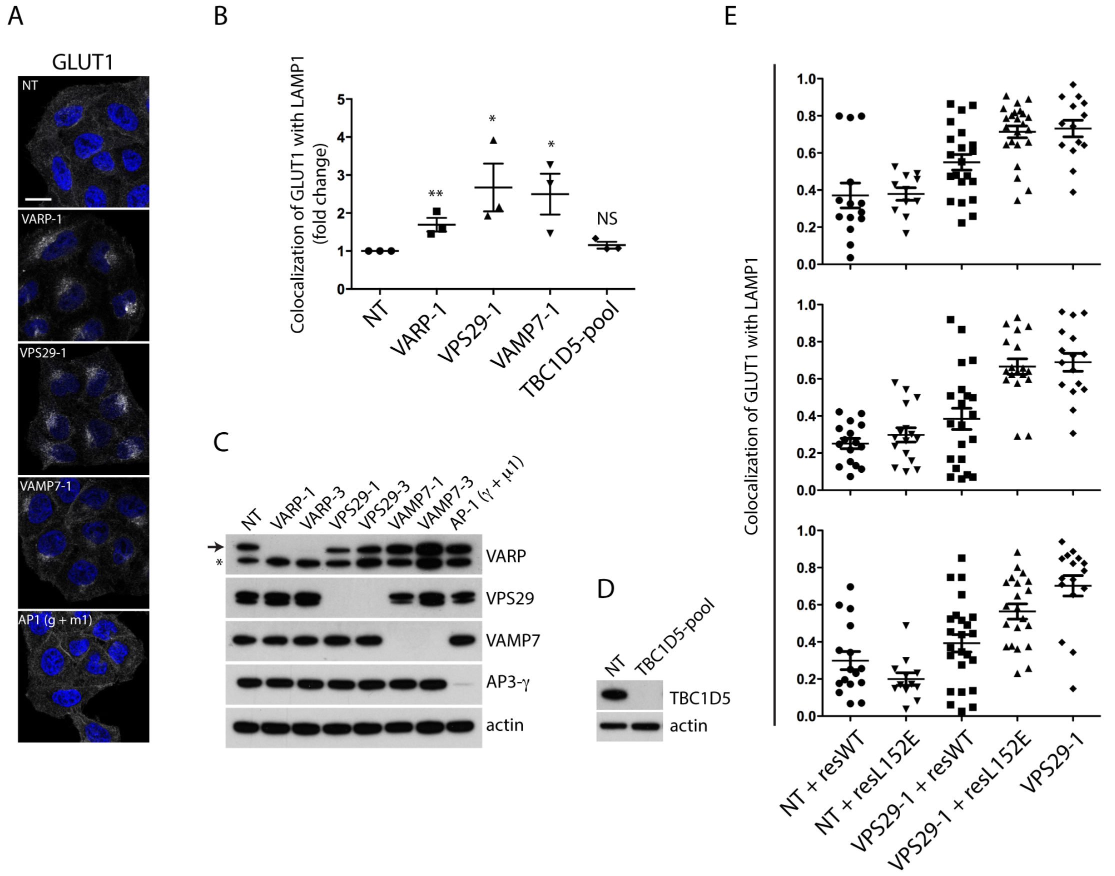

FIGURE S6

(A) Representative IF confocal microscopy of cells imaged in Figure 6A (GLUT1 (white), nuclei (blue)). Scale bar = 20μm.

(B) Fold-changes of colocalization coefficient measuring the colocalization of GLUT1 with LAMP1 after knock-down of VARP, VPS29, VAMP7 and TBC1D5. Mean (+/- SEM) of 3 experiments as described in Figure 6B and 6C (* = $p \leq 0.05$, **= $p < 0.02$, NS = not significant).

(C and D) Western blots confirming successful protein depletion after knock-down with siRNA oligonucleotides as indicated. * = non-specific band.

(E) Individual data points and means (+/- SEM) of LAMP1 vs GLUT1 colocalisation coefficients for the 3 individual experiments used for Figure 6D.

Table S1 Crystallographic data for VARP ANKRD1:Rab32SeMet(Q85L) and VARP ANKRD1:SeMetRab38(Q69L) crystals
Related to Table 1

	VARP:SeMet Rab32	VARP:SeMet Rab38
Space group	C2	C2
No. complexes in ASU	3	6
Unit cell (Å)	*a*=113.0 *b*=122.5, *c*=131.6	*a*=258.2, *b*=83.9, *c*=161.2
(˚)	β=113.25	β=122.42
Wavelength (Å)	0.9173	0.9795
Resolution range (Å)	57.05-2.97 (3.12-2.97)	80.19-4.50 (5.03-4.50)
Beamline	IO4-1	I03
Number of crystals	1	1
R_{merge}	0.385 (2.096)	0.370 (1.373)
R_{merge} in top intensity bin	0.061	0.049
R_{meas}	0.420 (2.279)	0.414(1.727)
R_{pim}	0.163 (0.883)	0.182 (0.756)
Number of total reflections	211443 (28839)	88684 (25413)
Number of unique reflections	33107 (4412)	17604 (4941)
Mean ([I]/sd[I])	7.7 (1.9)	4.7 (1.4)
Half-dataset correlation coefficient $CC_{1/2}$	0.964 (0.395)	0.978 (0.539)
Completeness (%)	97.4 (98.4)	99.8 (99.6)
Anomalous completeness (%)	96.6 (97.5)	99.2 (99.0)
Multiplicity	6.4 (6.5)	5.0 (5.1)
Anomalous multiplicity	3.2 (3.3)	2.6 (2.6)
D_{anom} Correlation between half-sets	-0.222 (inner 0.332)	0.046 (inner 0.508)
Wilson plot B ($Å^2$) [a]	38.4	108.5

$R_{merge} = \Sigma(I_{hl} - \langle I_h \rangle)/\Sigma \langle I_h \rangle$

$R_{meas} = \Sigma\sqrt{\ }(n_h/n_h-1)(I_{hl} - \langle I_h \rangle)/\Sigma \langle I_h \rangle$

$R_{pim} = \Sigma\sqrt{\ }(1/n_h-1)(I_{hl} - \langle I_h \rangle)/\Sigma \langle I_h \rangle$

[a] “Inner” resolution range 80.18 to 10.06 Å

Table S2 Proton Induced X-ray Emission data
Related to Figure 5

		Ca	Fe	Cu	Zn
ANKRD1	Metal atoms/ monomer	0.021±0.003	0.014±0.005	0.008±0.005	3.0±0.2
	av/MDL	4.2	6.2	1.2	296
ANKRD2	Metal atoms/ monomer	0.011±0.002	0.003±0.002	0.007±0.004	1.12±0.08
	av/MDL	1.9	1.2	1.7	95
Buffer	av/MDL	1.1	2.9	10.9	2.5

MicroPIXE results. The stoichiometry is calculated as an average of 4 individual measurements (Av). Errors on the number of atoms/protein monomer are 1 standard deviation from this average. Av/MDL (minimum detectable limit) indicates the significance of the result: <1, not present, 1-3, potentially present, >3, present at a methodologically significant level. The levels in the buffer indicate that the only detectable impurity (~ppm level) is Cu.

Supplementary Materials and Methods

DNA Constructs for crystallization and protein:protein interaction studies

All genes used in this study are human with the exception of Rab38 and retromer subunits, which were murine. Constructs used in this study: pMWVARP$_{1-1050}$His$_{10}$ WT and mutants, pGEX-6P1VARP$_{397-650}$His$_{6}$, pGEX-6P1VARP$_{421-650}$His$_{6}$, pGEX-6P1VARP$_{451-640}$His$_{6}$, WT and mutants, Q509A, L513D, K546D, Y550A, Q509A/Y550A, L513D/K546D, pGEX-6P1Rab38$_{1-188}$(Q69L mutant), pGEX-6P1Rab32$_{1-225}$ WT and mutants Q85L, T39N, Q85L/M91S/R91S, Q85L/G89T/N90S/V94L, Q85L/V100M/Q153M/V158M/I192M, pGEX-6P1Rab5$_{1-215}$ (Q79L mutant), pMWRab7$_{1-204}$(Q67L mutant), pGEXRab7L1$_{1-203}$(Q67L). pGEX4T2VPS29, pGEX4T2VPS35+Vps29, pMWHis$_{6}$VPS26, pGEX4T2VPS35+His$_{6}$VPS26, pGEX6P1 VARP 396-460, pGEX6P1 VARP 692-746 pGBT9 VPS26A, pGBT9 VPS26B, pGBT9VPS29 (WT, L152E, I91S), pGBT9 VPS35, pGAD-CVARP (1-1050, 1-135, 1-410, 1-450, 369-450, 400-450, 410-450, 692-730, 1-1050(HL1), 1-1050(HL2), 1-1050(HL1+HL2), 410-450(HL1), 692-730(HL2), 410-450(4C1), 692-730(4C2))

DNA constructs used for cell biology

pEGFP-N3 VARP-EGFP and pLXIN VARP-EGFP (WT, Q509A/Y550A(QY), L513D/K546D(LK), M684D/Y687S(MY), Q509A/Y550A/M684D/Y687S(QY+MY), C431S/C435S/C437S/C440S(4C1), C711S/C715S/C717S/C720S(4C2), C431S/C435S/C437S/C440S/C711S/C715S/C717S/C720S(4C1+4C2), H432S/L434A(HL1), H712S/L714A(HL2), H432S/L434A/H712S/L714A(HL1+HL2))

pTagRFP-N VPS29-TagRFP (WT, L152E), pIRESneo2 VPS29-EGFP (WT, L152E), pIRESneo2 EGFP-VPS35, pLXIN VAMP7-2xHA (WT, D69A/E71F/S73D)

Full crystallization and structure determination

Crystals of $VARP_{451-640}His_6$: $Rab32_{1-225}$(Q85L) and of VARP-ANKR domain 1:SeMet- $Rab32_{1-225}$(Q85L/V100M/Q153M/V158M/I192M) were grown by sitting drop vapor diffusion at 18˚C against well solutions containing 14-19% (w/v) of PEG 3350, 200 mM sodium citrate pH 8.0 at an initial complex concentration of 2.5mg/ml. Xe-derivatized crystals were produced by placing crystals in a sealed chamber into which xenon was forced to 10–15 atmospheres pressure for 1-2 min. Small crystals of $VARP_{451-640}His_6$:SeMet-$Rab38_{1-188}$(Q69L) complex were grown by sitting drop vapor diffusion at 18°C from complex at a concentration of 3.8 mg/ml against a reservoir containing 16% (w/v) of PEG 3350 and 200 mM sodium citrate pH 7.0. Larger crystals were grown by streak seeding.

All crystals were cryoprotected in their mother liquor containing 25 % (v/v) glycerol and flash cooled in liquid nitrogen. Diffraction data were collected at 100 K at Diamond Light Source plc. Crystals exhibited anisotropic diffraction and suffered severe radiation damage. Complete data sets often required collection of several data sets from different regions of the crystals Tables S1 and S2.

Datasets were indexed and integrated with Mosflm (Leslie, 2006), and scaled and reduced with Aimless (Evans, 2011). VARP-ANKR domain 1:Rab32 (Q85L) crystals belonged to the space group $P3_221$ and diffracted in the best directions to 2.8Å. The structure was solved by the Molecular Replacement with

Phaser (McCoy et al., 2007) using models based on the structures of Rab7A (PDB 1YHN) and the VARP ANKRD2 (PDB 4B93). A single solution was obtained with 3 molecules of complex per asymmetric unit with electron density maps exhibiting good connectivity for the three complexes and clear density for GppCp:Mg^{++}. Xe sites were calculated using Phaser and the anomalous phases used as restraints in initial rounds of refinement.

Crystals of VARP-ANKR domain 1:SeMet-Rab32 (Q85L) and of VARP-ANKR domain 1:SeMet-Rab38 (Q69L) both belonged to the space group C2, (see Table 1) and diffracted in their best directions to 3Å and 4.5Å respectively. Their structures were solved by Molecular Replacement using the coordinates of native VARP ANKRD1:Rab32(Q85L) complex as the search model. VARP ANKRD1:SeMet-Rab32(Q85L) consisted of three molecules of complex per asymmetric unit, whilst that of ANKR domain 1:SeMet-Rab38(Q69L) consisted of six molecules of complex per asymmetric unit. No further refinement of ANKR domain 1:SeMet-Rab38(Q69L) structure was undertaken due to the low resolution of the data.

Model building and refinement were carried out with Coot (Emsley et al., 2010) and Refmac (Murshudov et al., 2011) and the geometry of final models checked using MolProbity (Chen et al., 2010). Analysis of protein interfaces was performed with PISA (Krissinel and Henrick, 2007). Structure pictures were prepared with *PyMOL* [HTTP://WWW.PYMOL.ORG/].

Antibodies

GFP (IF: mAb and pAb, Molecular Probes, IP: anti-GFP pAb (Seaman et al., 2009)), VARP (pAb (Schafer et al., 2012)), VPS29 (goat pAb, Abcam), Rab32 (rabbit pAb,

gift from Miguel Seabra), VPS35 (mAb B-5, Santa Cruz), VAMP7 (IF: anti longin domain pAb (Pryor et al., 2008)), IB: mAb, gift from Andrew Peden), TBC1D5 (mAb E-9, Santa Cruz), FAM21 (pAb S-13, Santa Cruz), Tubulin (mAb DM1A, Sigma), Actin (rabbit pAb, Sigma), VPS26 (pAb (Seaman, 2004)), AP-1 γ-subunit (mAb 100.3, gift from Margaret S. Robinson), GLUT1 (pAb 15309, Abcam).

siRNA mediated gene knock-down

siRNA oligonucleotides targeting human genes were designed and synthesized by Dharmacon/Thermo Scientific. Cells were transfected (two transfections separated by 48hrs) using Oligofectamine (Life Technologies) according to manufacturer's instructions. On-Target Plus oligonucleotide catalogue numbers were as follows: non-targeting (NT) control (D-001810-01), VARP-1 (J-014788-09), VARP-3 (J-014788-11), VPS29-1 (J-009764-09), VPS29-3 (J-009764-11), VPS35-1 (J-010894-05), VPS35-2 (J-010894-06), VAMP7-1 (J-020864-05), VAMP7-3 (J-020864-07), Rab32-1 (J-009920-06), Rab32-2 (J-009920-07), TBC1D5 SMARTpool (L-020775-01), AP-1-γ SMARTpool (L-019183-00), AP-1-μ1 SMARTpool (L-013196-00).

Analytical Gel Filtration

Samples of reconstituted VARP-ANKR domain 1:Rab32 (Q85L) complex were run on a Superdex 75 PC3.2/30 using an AKTA Micro chromatography system. 50 μl protein samples at ten different concentrations varying from 0.1 μM to 150 μM were injected onto the column. Elution profiles were followed by measuring absorbance at 280nm and 230nm. The values of estimated molecular

weight and protein concentration in the peak were fitted to a saturation-binding curve using the program Prism.

Proton-induced X-ray emission

Proton-induced X-ray emission (PIXE) is an analytical tool for the unambiguous identification of atoms in a sample from the unique energy of their fluorescent X-rays, emitted on bombardment of the sample by energetic protons. These X-rays are detected in a lithium-drifted silicon detector with high-energy resolution. Stoichiometric quantitation is achieved for proteins by taking the ratio of the counts in the X-ray spectrum due to sulphur (originating from the known number of cysteines and methionines in the sequence) to the counts of the element of interest, in this case zinc (Garman and Grime, 2005; Garman and Zeldin, 2013).

Samples for analysis were gel filtered at 10 mg/ml into 20mM Tris pH 7.4, 100mM NaBr and 0.5mM TCEP in order to ensure low chlorine content and no non-protein sulphur. The low chlorine content is necessary because if the chlorine peak is large, its tail overlaps with the neighbouring peak from sulphur. This has the effect of increasing the lower limit of detection for sulphur. 0.2 μl of each sample was placed onto a 4 μm thick prolene film, and left to dry overnight. For the microPIXE analysis, a 2.5 MeV proton beam of diameter 2 μm was used and was scanned across the drop in the x and y directions. The resulting X-ray spectra were sorted into 2-D elemental maps. Both sulphur and zinc were clearly detectable and allowed the protein to be located for the selection of 4 suitable points for measurements from the sulphur (protein) rim of the map. The resulting spectra were analyzed in OMDAQ (Grime and Dawson, 1994). Sulphur-

normalisation was performed for all metals with potentially significant values, and the means and standard deviations for the four point measurements on each sample were calculated. Metal signals were deemed significant if the ratio of peak counts to minimum detectable limit (MDL), as determined by GUPIX (Maxwell et al., 1989), was >3, and of interest, but not significant, if between 1 and 3. The results are shown in Table S2.

Supplementary References

Chen, V.B., Arendall, W.B., 3rd, Headd, J.J., Keedy, D.A., Immormino, R.M., Kapral, G.J., Murray, L.W., Richardson, J.S., and Richardson, D.C. (2010). MolProbity: all-atom structure validation for macromolecular crystallography. Acta Crystallogr D Biol Crystallogr *66*, 12-21.

Emsley, P., Lohkamp, B., Scott, W.G., and Cowtan, K. (2010). Features and development of Coot. Acta Crystallogr D Biol Crystallogr *66*, 486-501.

Evans, P.R. (2011). An introduction to data reduction: space-group determination, scaling and intensity statistics. Acta Crystallogr D Biol Crystallogr *67*, 282-292.

Garman, E.F., and Grime, G.W. (2005). Elemental analysis of proteins by microPIXE. Progress in biophysics and molecular biology *89*, 173-205.

Garman, E.F., and Zeldin, O.B. (2013). Elemental Analysis of Proteins by Proton Induced X-ray Emission (microPIXE). Advancing Methods for Biomolecular Crystallography 79–89.

Grime, G.W., and Dawson, M. (1994). A PC-based data acquisition package for nuclear microbeam systems. Nuclear Instruments and Methods in Physics Research Section B *89*, 223-228.

Krissinel, E., and Henrick, K. (2007). Inference of macromolecular assemblies from crystalline state. J Mol Biol *372*, 774-797.

Leslie, A.G. (2006). The integration of macromolecular diffraction data. Acta Crystallogr D Biol Crystallogr *62*, 48-57.

Maxwell, J., Campbell, J., and Teesdale, W. (1989). The Guelph PIXE software package. Nuclear Instruments and Methods in Physics Research Section B *43*, 218-230.

McCoy, A.J., Grosse-Kunstleve, R.W., Adams, P.D., Winn, M.D., Storoni, L.C., and Read, R.J. (2007). Phaser crystallographic software. J Appl Crystallogr *40*, 658-674.

Murshudov, G.N., Skubak, P., Lebedev, A.A., Pannu, N.S., Steiner, R.A., Nicholls, R.A., Winn, M.D., Long, F., and Vagin, A.A. (2011). REFMAC5 for the refinement of macromolecular crystal structures. Acta Crystallogr D Biol Crystallogr *67*, 355-367.

Pryor, P.R., Jackson, L., Gray, S.R., Edeling, M.A., Thompson, A., Sanderson, C.M., Evans, P.R., Owen, D.J., and Luzio, J.P. (2008). Molecular basis for the sorting of the SNARE VAMP7 into endocytic clathrin-coated vesicles by the ArfGAP Hrb. Cell *134*, 817-827.

Schafer, I.B., Hesketh, G.G., Bright, N.A., Gray, S.R., Pryor, P.R., Evans, P.R., Luzio, J.P., and Owen, D.J. (2012). The binding of Varp to VAMP7 traps VAMP7 in a closed, fusogenically inactive conformation. Nat Struct Mol Biol *19*, 1300-1309.

Seaman, M.N. (2004). Cargo-selective endosomal sorting for retrieval to the Golgi requires retromer. J Cell Biol *165*, 111-122.

Seaman, M.N., Harbour, M.E., Tattersall, D., Read, E., and Bright, N. (2009). Membrane recruitment of the cargo-selective retromer subcomplex is catalysed by the small GTPase Rab7 and inhibited by the Rab-GAP TBC1D5. J Cell Sci *122*, 2371-2382.
