# Supplementary material for: VARP Is Recruited on to Endosomes by Direct Interaction with Retromer, Where Together They Function in Export to the Cell Surface
Source: Dev Cell. 2014 Jun 9;29(5):591–606. doi: 10.1016/j.devcel.2014.04.010 (PMC4059916; doi:10.1016/j.devcel.2014.04.010)
Supplement: Document S2. Article plus Supplemental Information [file mmc2.pdf]

Developmental Cell
Article

VARP Is Recruited on to Endosomes by Direct Interaction with Retromer, Where Together They Function in Export to the Cell Surface

Geoffrey G. Hesketh,[1,4,5] Inmaculada Pérez-Dorado,[2,4,6] Lauren P. Jackson,[1,7] Lena Wartosch,[1] Ingmar B. Schäfer,[2,8] Sally R. Gray,[1] Airlie J. McCoy,[1] Oliver B. Zeldin,[3] Elspeth F. Garman,[3] Michael E. Harbour,[1] Philip R. Evans,[2] Matthew N.J. Seaman,[1,*] J. Paul Luzio,[1,*] and David J. Owen[1,*]

[1]Cambridge Institute for Medical Research and Department of Clinical Biochemistry, University of Cambridge, Wellcome Trust/MRC Building, Cambridge Biomedical Campus, Hills Road, Cambridge CB2 0XY, UK
[2]Medical Research Council Laboratory of Molecular Biology, Francis Crick Avenue, Cambridge Biomedical Campus, Cambridge CB2 0QH, UK
[3]Department of Biochemistry, University of Oxford, South Parks Road, Oxford OX1 3QU, UK
[4]Co-first author
[5]Present address: Lunenfeld-Tanenbaum Research Institute, Mount Sinai Hospital, Toronto, ON M5G 1X5, Canada
[6]Present address: Department of Life Sciences, Imperial College London, Exhibition Road, South Kensington SW7 2AZ, UK
[7]Present address: Department of Biological Sciences, Vanderbilt University, 21st Avenue South, Nashville, TN 37232, USA
[8]Present address: Department of Structural Cell Biology, Max Planck Institute of Biochemistry, Am Klopferspitz 18, 82152 Martinsried, Germany
*Correspondence: mnjs100@cam.ac.uk (M.N.J.S.), jpl10@cam.ac.uk (J.P.L.), djo30@cam.ac.uk (D.J.O.)
http://dx.doi.org/10.1016/j.devcel.2014.04.010
This is an open access article under the CC BY license (http://creativecommons.org/licenses/by/3.0/).

SUMMARY

VARP is a Rab32/38 effector that also binds to the endosomal/lysosomal R-SNARE VAMP7. VARP binding regulates VAMP7 participation in SNARE complex formation and can therefore influence VAMP7-mediated membrane fusion events. Mutant versions of VARP that cannot bind Rab32:GTP, designed on the basis of the VARP ankyrin repeat/Rab32:GTP complex structure described here, unexpectedly retain endosomal localization, showing that VARP recruitment is not dependent on Rab32 binding. We show that recruitment of VARP to the endosomal membrane is mediated by its direct interaction with VPS29, a subunit of the retromer complex, which is involved in trafficking from endosomes to the TGN and the cell surface. Transport of GLUT1 from endosomes to the cell surface requires VARP, VPS29, and VAMP7 and depends on the direct interaction between VPS29 and VARP. Finally, we propose that endocytic cycling of VAMP7 depends on its interaction with VARP and, consequently, also on retromer.

INTRODUCTION

VARP (VPS9-domain ankyrin repeat protein) is a widely expressed, multidomain protein of 1,050 residues present in most animals (although lost from *D. melanogaster* and *C. elegans*), as well as in some species of unicellular organisms closely related to animals, including Capsaspora and Choanoflagellates. VARP is a Rab32/38 effector, and, in line with other Rab:Rab-effector systems, it has been assumed that Rab32 would be primarily responsible for recruiting VARP on to membranes (Tamura et al., 2009; Wang et al., 2008). VARP also possesses Rab21 GEF (guanine nucleotide exchange factor) activity (Zhang et al., 2006) and binds to VAMP7 (Figure 1A), an R-SNARE implicated in a variety of endocytic and exocytic fusion events (Bal et al., 2013; Fader et al., 2009; Monteiro et al., 2013; Moreau et al., 2011; Pryor et al., 2004; Rao et al., 2004) (reviewed in Chaineau et al., 2009). VARP binds to VAMP7 in its "closed," prefusion conformation, and this interaction inhibits VAMP7-mediated SNARE complex formation by clamping its regulatory longin domain on to its SNARE motif (Schäfer et al., 2012). Through associations with GolginA4 and Kif5A, VARP has also been suggested to participate in a molecular network that regulates delivery of VAMP7-containing vesicles toward the cell periphery (Burgo et al., 2012), which could contribute to the process of neurite outgrowth (Burgo et al., 2009).

The Rab family of small GTPases plays a key role in protein sorting and membrane trafficking throughout eukaryotic cells (reviewed in Stenmark, 2009). Rab32 is an endosomal Rab GTPase, thought to have been present in the last eukaryotic common ancestor, with Rab38 arising in vertebrates as an evolutionary gene duplication of Rab32 (Klöpper et al., 2012). Rab32 (together with Rab7A and B, Rab7L1, Rab9A and B, and Rab23) is classified in the late endocytic group III Rabs (Klöpper et al., 2012), a group of Rabs with functions at multiple points along the endocytic system. For example, Rab7A mediates the recruitment of the retromer complex (Rojas et al., 2008; Seaman et al., 2009) and is also important for delivery to lysosomes (reviewed in Stenmark, 2009).

Rab32 is expressed in a variety of cell types (Cohen-Solal et al., 2003) and has been implicated in the biogenesis of, and

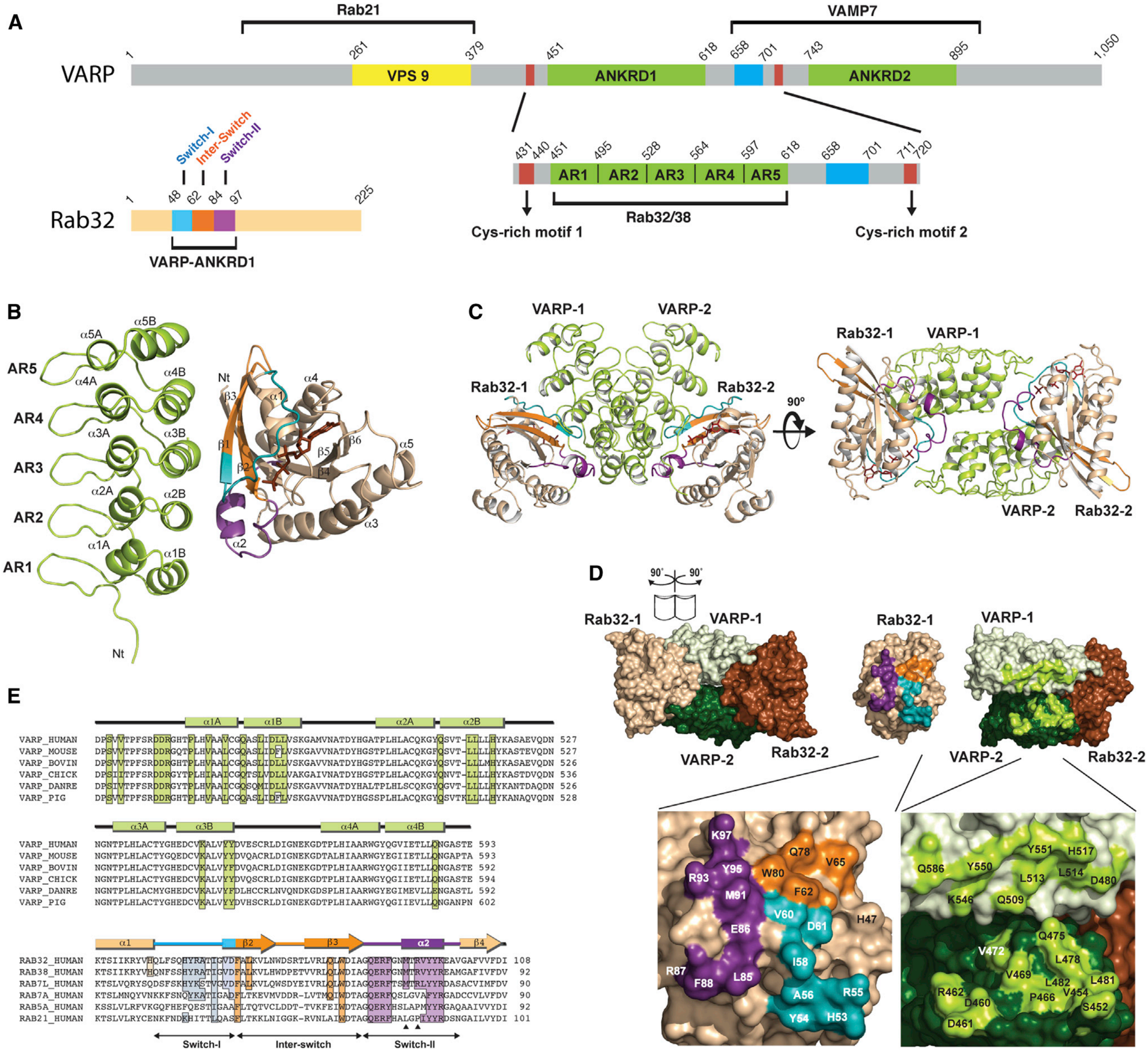

Figure 1. Structure of the VARP-ANKRD1:Rab32 Complex

(A) Schematic representation of VARP and Rab32 proteins, highlighting the functions of different domains. The domain color scheme depicted is used in all subsequent figures.

(B) Structure of the VARP (residues 451–640): Rab32(Q85L) heterodimer in "ribbon representation," with GppCp shown as ball and stick.

(C) Orthogonal views of a VARP:Rab32 2:2 cross-dimer formed from two heterodimers that are in different asymmetric units. In the left-hand view, the membrane is located at the bottom, and in the right-hand view, the tetramer is viewed "through the membrane."

(D and E) Surface representation, in (D), of the cross-dimer "opened out" as indicated to show the residues involved in the two different VARP:Rab32 interactions. The main residues involved are labeled in the zoomed-in views (bottom panels) and are shown highlighted in the same colors in the sequence alignment shown in (E).

See also Figure S1.

transport to, lysosome-related organelles (LROs) such as melanosomes (Bultema et al., 2012; Park et al., 2007; Tamura et al., 2009; Wasmeier et al., 2006), autophagosomes (Wang et al., 2012), and platelet dense granules (Ambrosio et al., 2012). Rab38 is expressed in specialized cell types enriched in LROs (Osanai et al., 2005) and serves partially redundant functions with Rab32. Consistent with VARP functioning as a Rab32/38 effector, in melanocytes, VARP colocalizes with Rab32/38 on endocytic organelles and melanosomes (Wasmeier et al., 2006). In other cell types, VARP localizes primarily to endosomes but is also present on vesicular/tubular clusters, lysosomes, and the plasma membrane (Schäfer et al., 2012), a pattern of localization consistent with VARP's multiple functions.

We set out to determine the molecular mechanism of the expected recruitment of VARP to endosomal membranes by Rab32/Rab38. We determined the crystal structure of the complex between the first ankyrin repeat domain of VARP with the GTP hydrolysis-deficient forms of Rab32 and Rab38. Unexpectedly, however, we found that mutations based on these structures, which abolished their interaction in vitro, did not prevent endosomal recruitment of VARP in vivo. Subsequently, we have identified the retromer subunit VPS29 as a direct binding partner of VARP. Precise mapping of the VPS29-VARP interaction shows this interaction recruits VARP on to endosomes, and we demonstrate that VARP, retromer, and VAMP7 operate together in a pathway that traffics the known retromer cargo GLUT1 from endosomes to the cell surface.

RESULTS

Structure of the VARP Ankyrin Repeat-Containing Domain:Rab32 Complex

VARP has been shown to interact with Rab32/38 via its first ankyrin repeat-containing domain (Tamura et al., 2009, 2011; Wang et al., 2008). Two fragments of VARP comprising residues 397–650 and 451–640 of VARP, which contains the first ankyrin repeat-containing domain (ANKRD1; Figure 1A), bind to Rab32 in a GTP-dependent manner with a dissociation constant, K_D, of 7 ± 3 μM and a K_D of 2.5 ± 0.4 μM, respectively (Figure 2 and Figure S1A available online). We were able to crystallize a 1:1 complex of the smaller VARP fragment (residues 451–640) with the GTP hydrolysis-defective mutant of Rab32 (Q85L) in the presence of the nonhydrolyzable analog of GTP, GppCp. Its structure was solved at 2.8Å resolution by molecular replacement using VARP ANKRD2 and Rab7:GTP as search models (Figure 1B; Table 1). The final model comprises three molecules of the complex in the asymmetric unit, where VARP (residues 452–618), Rab32 (residues 22–198), and Rab32's bound GppCp:Mg^{2+} are well defined in the electron density map (Figures S1C and S1D). The three complexes in the asymmetric unit all have the same structure, with root-mean-square deviation (rmsd) values of 0.32 Å (for 306 Cα atoms of molecules A and B) and 0.33 Å (for 313 Cα atoms of molecules A and C).

VARP ANKRD1 contains five tandem ankyrin repeats that form a slightly curved stack (Figure 1B). The structure of Rab32 bound to GppCp:Mg^{2+} resembles that of other effector-bound Rabs (reviewed in Lee et al., 2009). The P loop (residues 32–39) stabilizes the Mg^{2+}-coordinated triphosphate moiety of the nucleotide, while Switch-I (residues 48–61) folds over the nucleotide molecule and stabilizes the γ-phosphate of the GppCp. Switch-II (residues 84–97) also stabilizes the γ-phosphate but differs somewhat in conformation as compared to those in other GTP-bound Rab structures (Figure S1B).

The VARP ANKRD1:Rab32 heterodimer buries 1,530 $Å^2$ of solvent accessible surface area (calculated with PISA; Krissinel and Henrick, 2007) (Figure 1D). The elongated interface consists of a central hydrophobic patch of residues from helices α2B (L513 and L514) and α3B (Y550 and Y551) of VARP and residues from Switch-I (V60), interswitch (F62, L69, and W80), and Switch-II (M91, V94, K97, and Y95) of Rab32. Flanking the hydrophobic core of the interface are two salt bridges formed through Switch-II of Rab32 (D480^{VARP}-R93^{Rab32}, H517^{VARP}-K97^{Rab32}) and a third one through the interswitch of the GTPase (K546^{VARP}-D61^{Rab32}). The residues involved in these interactions are conserved in other organisms (Figure 1E). Many of the key interacting residues in Rab32 are unique to Rab32/38, thus explaining the selectivity for VARP binding Rab32/38 over other endosomal Rabs, as demonstrated in "glutathione S-transferase (GST) pull-down" experiments (Figure S1E).

Interactions between the Switch-I/interswitch/Switch-II regions of Rab GTPases usually occur with two long α helices of an effector, which are often antiparallel and connected by a tight turn (Figure S2A). However, although also displaying a similar low micromolar K_D interaction to other Rab:Rab-effector interactions (Stenmark, 2009), the Rab-binding region of VARP is formed by residues from four short parallel α helices, making this an unexpected mechanism of Rab:Rab-effector binding. Most stacked ankyrin-repeat domains bind their partners on their concave side, whereas our results show that the interaction occurs on the convex side of the stack and, thus, leaving the concave side available to potentially bind other VARP interactors.

Mutational Analysis of the VARP-ANKRD1:Rab32 Complex Interface

Four residues within VARP that contribute to the complex interface, L513^{VARP}, Y550^{VARP}, Q509^{VARP}, and K546^{VARP} were mutated, and their ability to bind Rab32 in its GTP-locked state in solution was investigated (note that Q509^{VARP} and Y550^{VARP} have been previously proposed to participate in the VARP:Rab32/38 interaction; Tamura et al., 2011). Two double mutants, Q509A/Y550A and L513D/K546D, were also generated. The results of GST pull-down assays (Figure 2C) showed that VARP (wild-type [WT]) binds GSTRab32(Q85L) but not GST. VARP(Y550A) and VARP(K546D) showed reduced binding to GSTRab32:GTP, while the mutants Q509A, L513D, L513D/K546D, and Q509A/Y550A showed no detectable binding (Figure 2C). In broad agreement with these data, subsequent isothermal titration microcalorimetry (ITC) showed that the K_Ds for the interaction of these mutants were increased from 2.5 μM for WT VARP451-640 to ~8 μM for K546D, ~22 μM for Y550A, and ~30 μM for Q509A (Figure 2B). The L513D mutation reduced binding to below detectable levels (K_D > 300 μM), as did the double mutant Q509A/Y550A, but the L513D/K546D mutant was unstable in the constantly stirring environment of the ITC cell.

Mutating residues M91/R93 in Switch-II of Rab32, which participate in the interaction with VARP (Figure 2D), resulted in reduced binding as shown by pull down (Figure 2F) and no detectable binding by ITC (Figure 2E)

Dimerization of the Rab32:VARP Heterodimer

Many small GTPase:effector complexes are believed to form dimers, which increases both the apparent affinity and specificity of the recruitment of effectors to membrane-anchored GTPases (Lee et al., 2009; Panic et al., 2003). In the crystal, all VARP:Rab32 heterodimers form identical homodimers, resulting in the heterotetramers shown in Figures 1C and 1D. The majority of the heterodimer/heterodimer interface arises from the VARP molecules contacting the other Rab32 molecule in the tetramer through residues from Switch-I and Switch-II that are different

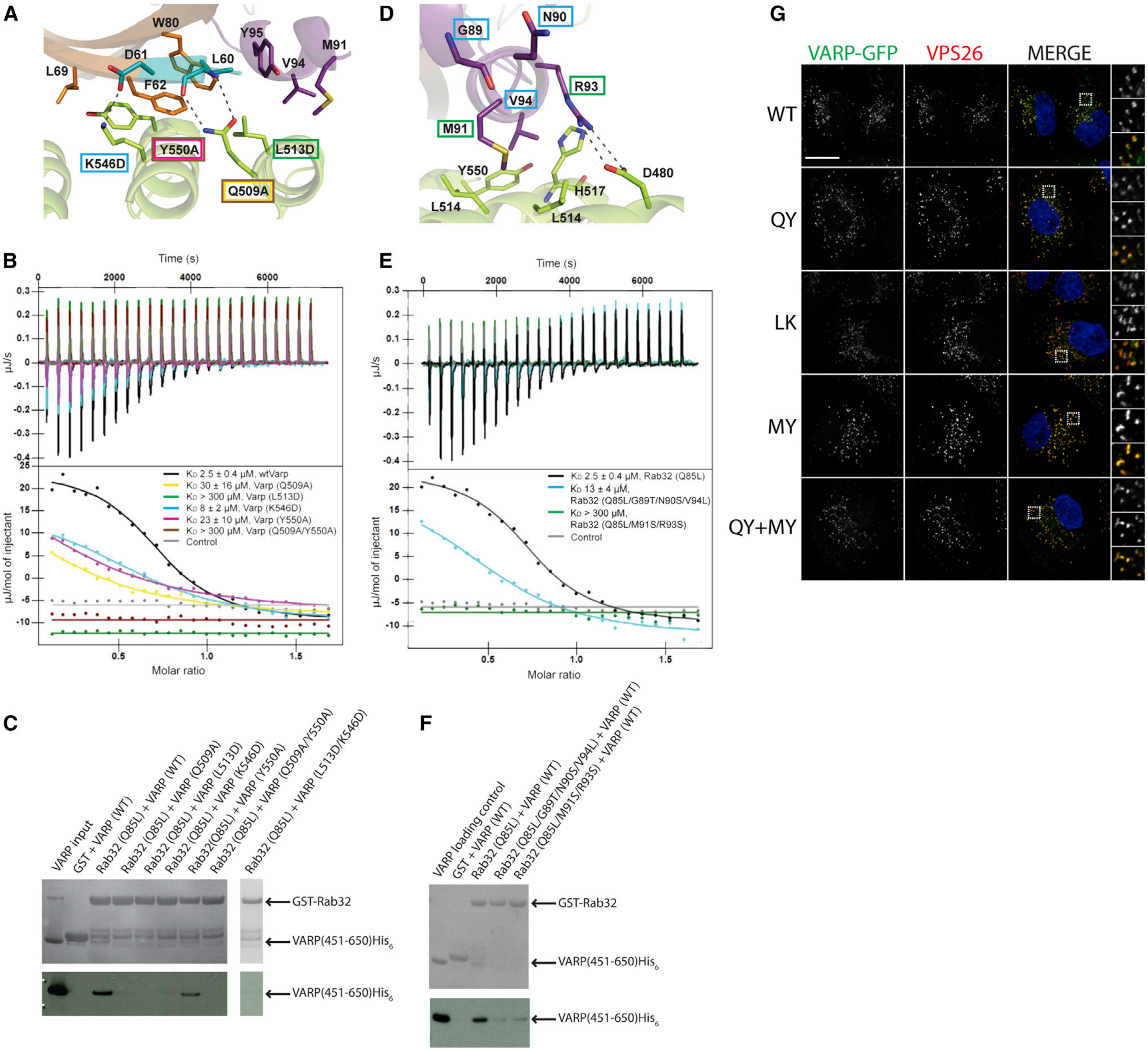

Figure 2. Biochemical Analysis and In Vivo Role of the VARP:Rab32 Interaction

(A) Molecular details of the VARP:Rab32 heterodimer interface. Residues mutated in VARP are boxed in color.

(B) Effects on the binding of the VARP:Rab32 interaction of mutating key VARP residues in the interface as measured by ITC. Curves are color coded as in (A).

(C) GST pull-downs using GSTRab32(Q85L) and VARP451-640His$_6$ showing the effect of mutating key residues on VARP in the interaction interface (A).

(D) Molecular details of VARP:Rab32 interface in orthogonal view to (A). Residues mutated in Rab32 are boxed in color.

(E) Effects on the VARP:Rab32 interaction of mutating key Rab32 residues in the interface as measured by ITC. Curves are color coded as in (D).

(F) GST pull-downs using GSTRab32(Q85L) and VARP451-640His$_6$ showing the effect of mutating key residues on Rab32 in the interaction interface (D).

(G) IF confocal microscopy of cytosol-extracted HeLa cells stably expressing VARP-GFP constructs: WT, Q509A/Y550A (QY), L513D/K546D (LK), M684D/Y687S (MY), and Q509A/Y550A/M684D/Y687S (QY+MY). GFP, green; VPS26, red; nuclei, blue, merged panel (MERGE). Boxed regions in the merged panels are shown as separate green (top), red (middle), and merged (bottom) channels on the right. Colocalization coefficients for the VPS26 versus GFP signals were as follows: WT, 0.89; QY, 0.75; LK, 0.59; MY, 0.66; QY+MY, 0.64. Scale bar, 20 μm.

In (B) and (E), curves for data showing binding are the mean of a minimum of three experiments ± SD. In (C) and (F), top panels are gel stained with Coomassie blue, and the lower panels are western blots using an anti-His probe. See also Figure S2.

from those involved in the VARP:Rab32 heterodimer formation. This second VARP:Rab32 interface buries 1,460 Å^2 of solvent accessible surface area, making the total solvent accessible buried surface area on tetramer formation around 6,400Å^2, which suggests that the tetramer should be stable. Existence of a dimer of heterodimers in solution is supported by analytical gel filtration, which is consistent with the existence of a concentration-dependent equilibrium of a 2:2 tetramer and a 1:1

Table 1. Crystallographic Data for VARP ANKRD1:Rab32(Q85L) Crystals

Parameter	Native	Xenon
Space group	$P3_221$	$P3_221$
Number of complexes in ASU	3	3
Unit cell (Å)	*a* = *b* = 144.4, *c* = 135.7	*a* = *b* = 144.9, *c* = 137.0
Wavelength (Å)	0.9763	1.7
Resolution range (Å)	72.22–2.80 (2.91–2.80)	72.45–3.30 (3.53–3.30)
Beamline	I03	I03
Number of crystals	3	1
R_{merge}	0.237 (2.215)	0.318 (2.091)
R_{merge} in top intensity bin	0.070	0.057
R_{meas}	0.256 (2.460)	0.328 (2.153)
R_{pim}	0.093 (1.042)	0.077 (0.508)
Number of total reflections	295,603 (21,370)	437,743 (78,174)
Number of unique reflections	40,077 (4,267)	25,238 (4,491)
Mean ([I]/SD[I])	8.8 (0.9)	9.8 (2.3)
Half-data set correlation coefficient $CC_{1/2}$	0.989 (0.229)	0.996 (0.663)
Completeness (%)	98.8 (94.1)	99.4 (98.9)
Anomalous completeness (%)		99.6 (99.1)
Multiplicity	7.4 (5.0)	17.3 (17.4)
Anomalous multiplicity		9.0 (8.9)
Δ_{anom} Correlation between half-sets		0.146 (inner 0.763)
Wilson plot B ($Å^2$)[a]	54.1	79.3
Refinement		
Number of atoms		
Protein	8,040	
Ligand	99	
Xe		9
H_2O	145	
R-factor	0.19	
R_{free}	0.24	
Number of reflections (number R_{free})	38,004 (2,011)	
<B > ($Å^2$)	74.2	
rms bond length deviation (Å)	0.011	
rmsd angle deviation (°)	1.589	
Ramachandran favored (%)	92.8	
Ramachandran outliers (%)	1.6	

$R_{merge} = \Sigma(I_{hl} - < I_h >)/\Sigma < I_h >$.
$R_{meas} = \Sigma\sqrt{(n_h/n_h - 1)}(I_{hl} - < I_h >)/\Sigma < I_h >$.
$R_{pim} = \Sigma\sqrt{(1/n_h - 1)}(I_{hl} - < I_h >)/\Sigma < I_h >$.
ASU, asymmetric units.
[a]"Inner" resolution range, 72.45–9.33 Å.

heterotetramer in solution (Figures S2B and S2C). Further evidence comes from size exclusion chromatography with multiangle light scattering (data not shown), as well as the observation that, in crystals, VARP ANKRD1:SeMet-Rab32Q85L and VARP ANKRD1:Rab38Q69L contain the same "cross-dimer of heterodimers" across multiple crystal forms, as compared to the wtVARP:Rab32Q85L complex (Tables 1 and S1).

VARP Recruitment to Endosomes Does Not Depend on Binding to Rab32/38 and/or VAMP7

It is generally accepted that most Rab effectors are recruited to their respective sites of action through binding to their cognate Rab (reviewed in Stenmark, 2009). Furthermore, taking into account the published literature and conceptual similarities to other Rab:Rab-effector interactions (such as binding to GTP-bound Rab only and Rab-induced dimerization), we anticipated that VARP endosomal recruitment would be Rab32/38 dependent. However, when stably expressed in HeLa cells, the Rab32/38 binding-deficient mutant versions of VARP-green fluorescent protein (GFP) (Q509A/Y550A and L513D/K546D) localized to punctate endosomal structures in a manner similar to that of WT VARP (Figures 2G and S2D). We further tested the possibility that VAMP7 could also be involved in VARP recruitment, but we observed that stably expressed mutant forms of VARP-GFP that cannot bind to VAMP7 (M684D/Y687S), or to Rab32/38 and VAMP7 simultaneously (Q509A/Y550A/M684D/Y687S), remained localized to endosomes. These data led us to conclude that the initial recruitment of VARP to endosomes is not dependent on its binding to either Rab32/38 or VAMP7 but must be dependent on another membrane-associated protein or a membrane component that interacted with VARP, which we set out to identify. Because we found that VARP recruitment to endosomes was not dependent on Rab32, we felt that further investigation of the exact function of this GTPase fell outside the scope of the present study.

VARP Is Recruited to Endosomes by Retromer

In separate experiments, it had been observed that native immunoprecipitation of VPS29-GFP or GFP-VPS35 followed by mass spectrometry identified VARP as a potential interacting partner of retromer (Figure S3A), in addition to established retromer accessory proteins, e.g., the WASH complex and the RabGAP TBC1D5 (Harbour et al., 2010; Seaman et al., 2009). Although far fewer VARP peptides than TBC1D5 and WASH complex peptides were isolated in these screening studies, the endosomal localization and central role in endocytic trafficking of retromer led us to consider it as a candidate for the role of recruiting VARP on to endosomes. In HeLa cells stably expressing VARP-GFP, small interfering RNA (siRNA)-mediated depletion of the retromer subunits VPS29 or VPS35, but not of Rab32 or VAMP7, resulted in loss of VARP-GFP but not of EEA1 recruitment to endosomes (Figures 3A and 3B; Figures S3B–S3D).

We next examined the subcellular colocalization of stably expressed VARP-GFP and the endogenous retromer and WASH complexes by immunofluorescence (IF) confocal microscopy. WT-VARP-GFP exhibited robust colocalization with the endogenous retromer subunit VPS26 on both vacuolar and tubular domains of endosomes (Figures 3C and 3D). The WASH complex subunit FAM21 (Harbour et al., 2012) colocalized with VARP and retromer on vacuolar domains of endosomes but was largely excluded from tubules (Figures 3C and S3E) (Harbour et al., 2012). The Rab5 effector EEA1 appeared

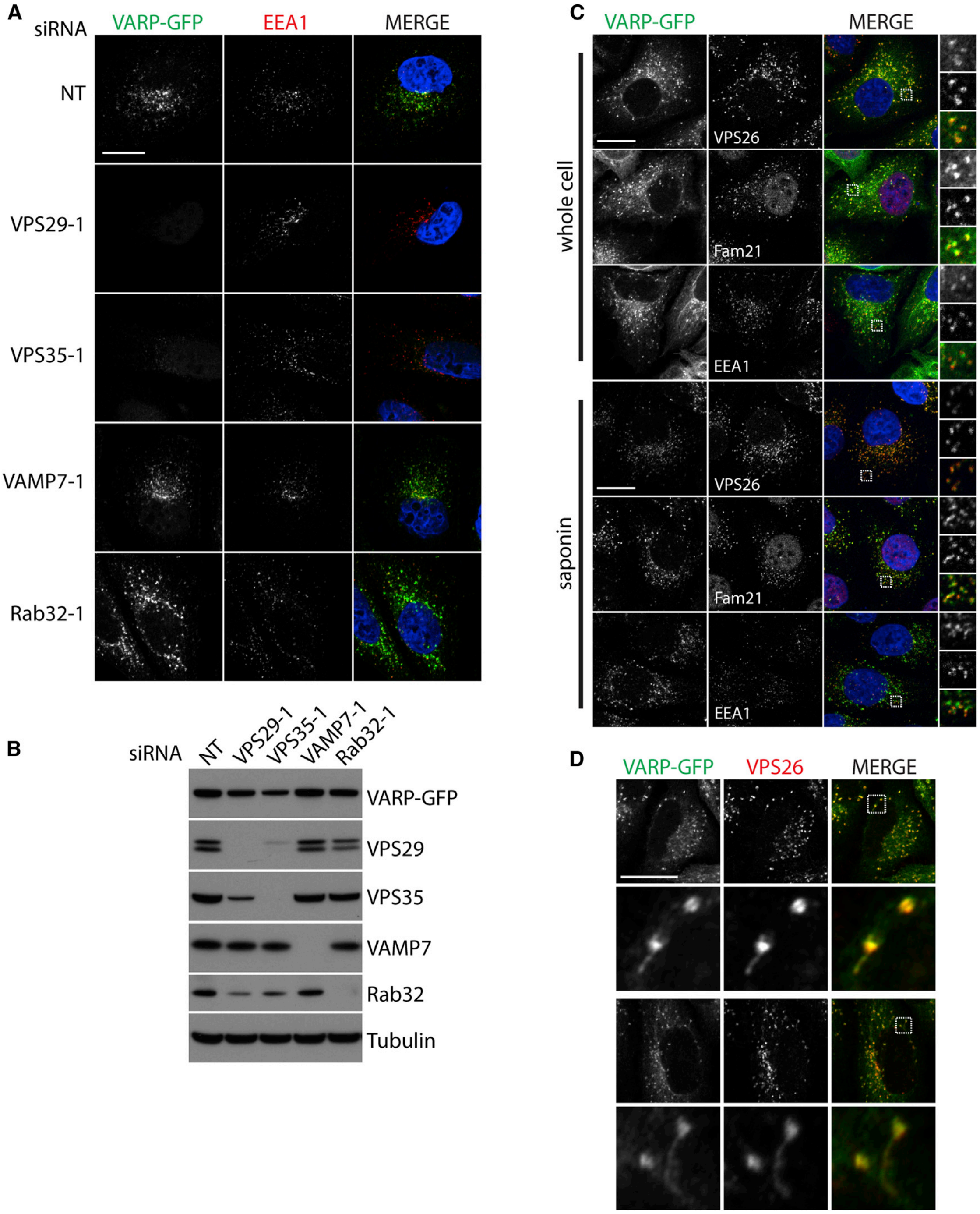

Figure 3. Retromer Recruits VARP on to Endosomes

(A) IF confocal microscopy of cytosol-extracted HeLa cells stably expressing VARP-GFP, which were knocked down using single siRNA oligonucleotides at 100 nM (NT, nontargeting control, VPS29-1, VPS35-1, VAMP7-1, Rab32-1). GFP, green; EEA1, red; nuclei, blue, merged panel (MERGE).

(B) Western blots of the cells imaged in (A) showing successful protein depletion.

(C) IF confocal microscopy of VARP-GFP cells. GFP, green; and either VPS26, Fam21, or EEA1, red; nuclei, blue, merged panels. Cells were either fixed intact (whole cell) or after cytosol extraction (saponin). Boxed regions in the merged panels are shown as separate green (top), red (middle), and merged (bottom) channels on the right. Colocalization coefficients for VPS26, FAM21, and EEA1 versus GFP were as follows, respectively (saponin only): VPS26, 0.56; FAM21, 0.16; EEA1, 0.67.

(D) IF confocal microscopy image of VARP-GFP cells. GFP, green; VPS26, red; the boxed regions (shown in the MERGE column) are enlarged below each image, demonstrating colocalization of VARP-GFP and VPS26 on both vacuolar and tubular domains of endosomes.

Scale bars, 20 μm. See also Figure S3.

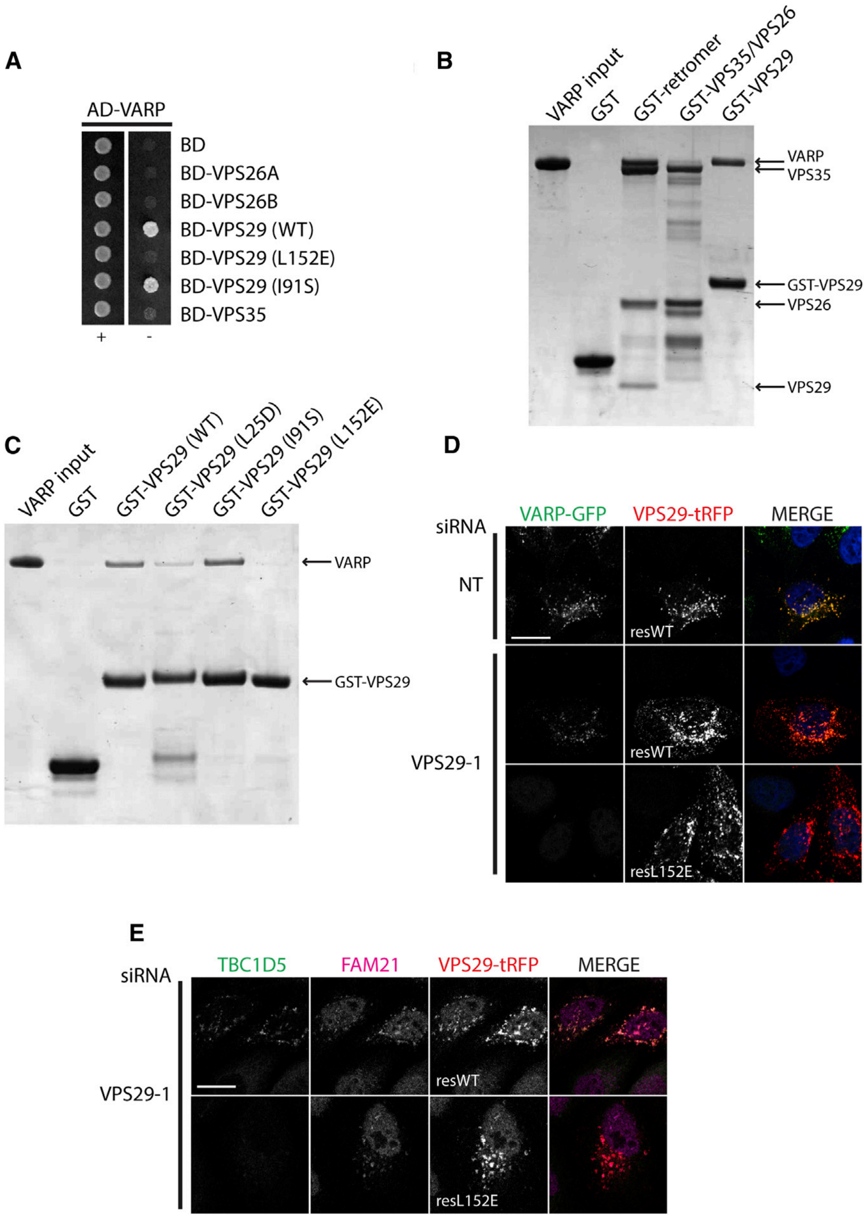

Figure 4. VARP Binds to a Conserved Hydrophobic Patch on VPS29

(A) Y2H analysis using the activation domain (AD) fused to VARP and the DNA binding domain (BD) fused to the indicated retromer subunits. Strains were grown in the absence (−) or presence (+) of histidine.

(B) Coomassie-stained gel of GST pull-down experiment showing the interaction of VARP-His_{10} (input, left lane) with GST alone, GST-VPS35 + VPS26 + VPS29 (GST-retromer), GST-VPS35 + VPS26 (GST-VPS35/VPS26), and GST-VPS29.

(C) Coomassie-stained gel of GST pull-down experiment showing the interaction of VARP-His_{10} (input, left lane) with GST alone, GST-VPS29 WT, and L25D, I91S, and L152E mutants.

(D) IF confocal microscopy of cytosol-extracted VARP-GFP cells knocked down using single siRNA oligonucleotides at 100 nM (NT, non-targeting control, VPS29-1). Twenty-four hours prior to fixation, cells were transiently transfected with VPS29-tRFP WT or L152E mutant. MERGE, merged panels.

(E) IF confocal microscopy of cytosol-extracted HeLa cells following knockdown with 100 nM VPS29-1 siRNA oligonucleotide performed as in (D). Twenty-four hours prior to fixation, cells were transiently transfected with VPS29-tRFP WT or L152E mutant (TBC1D5 [green], FAM21 [magenta], VPS29-tRFP imaged by native fluorescence, and nuclei [blue, merged panels]). Scale bars, 20 μm.

See also Figure S4.

to localize in many instances to the same endosomes as VARP, although it was differentially partitioned on those structures when compared with VARP, which colocalizes with retromer and the WASH complex (Figures 3A, 3C, and S3F).

Identification and Characterization of the VARP:VPS29 Interaction

We used a combination of yeast 2-hybrid (Y2H) analysis, GST pull-downs with bacterially expressed proteins, and in vivo siRNA knockdown and rescue experiments to identify the retromer subunit VPS29 as the direct binding partner of VARP. By Y2H, strains expressing both VARP and VPS29 exhibited growth, but no growth was observed when VARP was coexpressed with retromer subunits VPS26A, VPS26B, or VPS35 (Figure 4A). In agreement with these data, in GST pull-downs, full-length VARP bound to GST-VPS29 and a retromer subcomplex containing GST-VPS35, VPS26, and VPS29 with near-1:1 stoichiometry but not to a retromer subcomplex of GST-VPS35 and VPS26 (Figure 4B). The interaction was further mapped, using fully folded (Collins et al., 2005) mutant versions of VPS29 in both Y2H analysis and GST pull-downs, to a hydrophobic patch on the surface of VPS29, centered on Leu152 (Figures 4A, 4C, and S4A), which also binds to the RabGAP TBC1D5 (Harbour et al., 2010) and, possibly, very weakly to Snx1 (Collins et al., 2005; Swarbrick et al., 2011). In HeLa cells, endogenous VARP was coimmunoprecipitated with stably expressed WT-VPS29-GFP but not with L152E-VPS29-GFP (Figure S4B). In vivo, the loss of endosomal recruitment of VARP-GFP following VPS29 depletion was rescued by expression of an siRNA-resistant form of VPS29-TagRFP (red fluorescent protein) (Figures 4D and S4C). Consistent with the Y2H and biochemical interaction data, recruitment of VARP-GFP (or TBC1D5; Figures 4E and S4D) was not rescued on expression of the L152E-VPS29 mutant. The specificity of this effect was demonstrated by the fact that FAM21 localization, which was also lost from endosomes on VPS29 depletion due to destabilization of the retromer complex (Figure S4D), was rescued by both WT and L152E forms of VPS29 (Figure 4E), since FAM21 associates with retromer by binding to VPS35 (Harbour et al.,

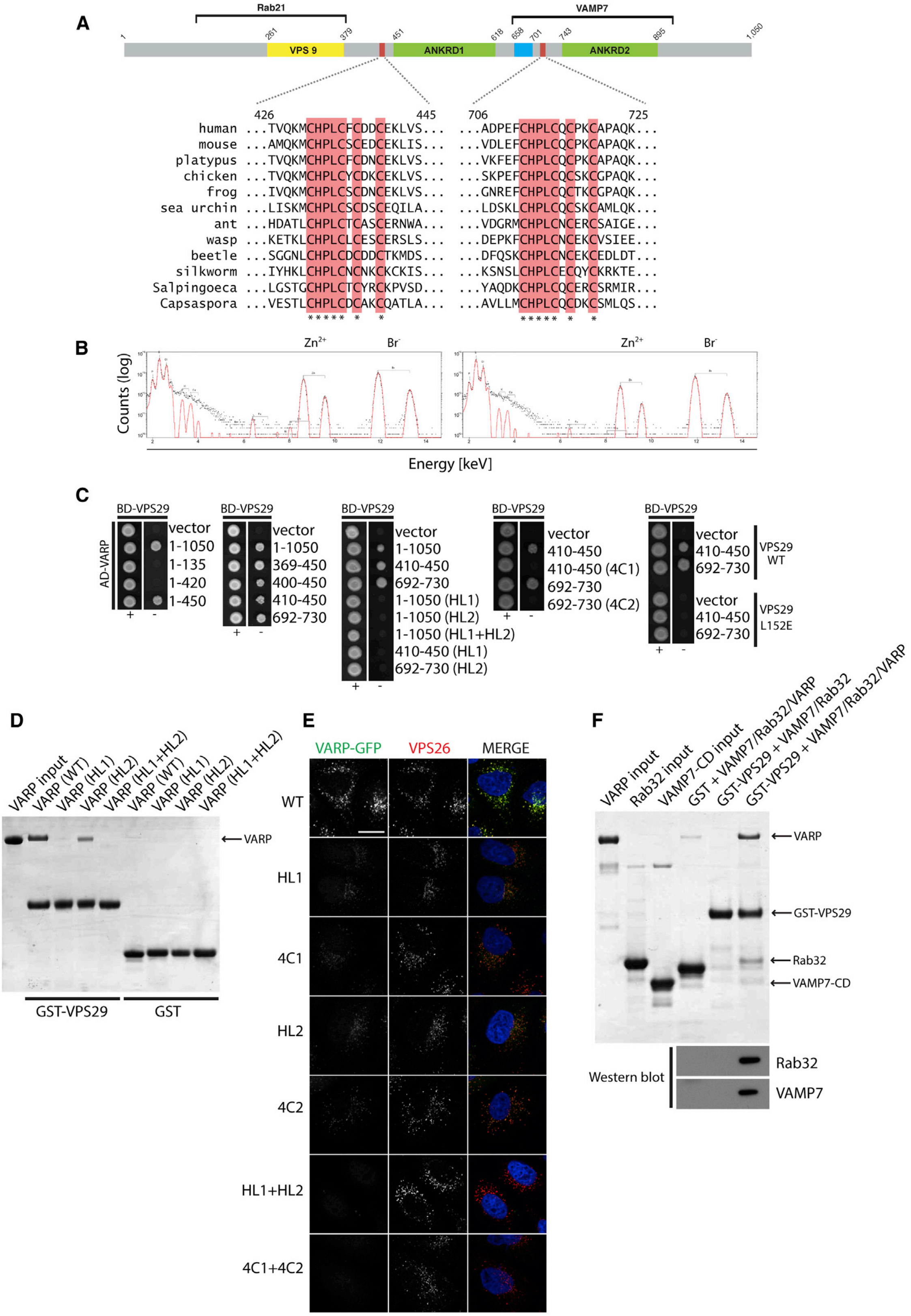

(legend on next page)

2012; Helfer et al., 2013; Jia et al., 2012). Furthermore, the endosomal recruitment of EEA1 was not disrupted upon VPS29 depletion, indicating that VPS29 depletion does not simply disrupt overall endosomal integrity (Figure 3A; Figures S3B and S3D). Thus, we conclude that VARP is recruited to endosomes through direct interaction with VPS29.

VPS29 Interacts with Two Cysteine-Rich Motifs within VARP

The evolution of VARP appears to have involved tandem duplication of a region containing six ankyrin repeats. Two regions of VARP (residues 410–450 and 692–730), which separate the first two ankyrin repeats, each contain a cysteine-rich consensus motif with the sequence CHPLCxCxxC that is conserved across all species (Figure 5A). Each region was shown independently to bind directly to VPS29 by Y2H (Figure 5C). Indeed, in ITC experiments, recombinant VARP fragments containing the Cys-rich motifs (residues 396–460 and 692–746) in isolation were able to bind to VPS29 with K_Ds in the low micromolar range (~13 μM and ~5 μM, respectively; Figure S5A).

The presence of a spatially conserved pattern of cysteines in an intracellular protein suggests the involvement of a metal: most likely, zinc. Consequently, elemental analysis by microbeam proton-induced X-ray emission (microPIXE) (Garman and Grime, 2005) was carried out on both bacterially expressed domains to investigate this possibility. The resulting data (Figure 5B and Table S2) indicate stoichiometric levels of Zn^{2+} in both Cys-rich motifs and that no other nonorganic elements are present at levels above 0.02 atoms per motif. Attempts to abrogate the interaction between VARP and VPS29 in vitro by adding EDTA in an effort to chelate the bound Zn^{2+} failed, presumably because of the difficulty of extracting tightly bound Zn^{2+}, as has been previously observed for other "double Zn-finger"-containing proteins (J. Deane, personal communication).

Mutation of all four Cys residues to Ser (4C1 or 4C2), or of the conserved His and Leu residues to Ser and Ala (HL1 or HL2), in the context of ~40-residue fragments containing the first or the second Cys-rich motifs, respectively, abolished binding to VPS29 by Y2H (Figure 5C). In ITC experiments using similar fragments, the HL2 mutation reduced binding to undetectable levels, while the 4C2 mutation decreased binding by ~20-fold (Figure S5A). In the context of full-length VARP, the HL1 and HL2 mutations reduced binding to VPS29 to below-detectable levels by Y2H (Figure 5C). However, in GST pull-downs, only simultaneous mutation of both HL1 and HL2 fully abolished VARP binding to VPS29 (Figure 5D). This agrees with data from HeLa cells stably expressing VARP-GFP, where extraction of non-membrane-associated proteins by a brief saponin wash prior to fixation resulted in only a partial loss of VARP-GFP from endosomes upon mutation of individual Cys-rich motifs, whereas simultaneous mutation of both motifs led to complete loss of VARP-GFP from endosomes (Figure 5E). This is in contrast to the effect of simultaneously mutating Rab32 and VAMP7 binding sites within VARP (Figure 2G). When these data are considered along with the small size of the VPS29 hydrophobic patch centered on L152 (Figure S4), the relative band intensities in Figures 4B and 5D, and the observation that a single Cys-rich motif binds to VPS29 with low micromolar K_D affinity (Figure S5), the most likely scenario for the interaction between full-length VARP and VPS29 is a 1:1 stoichiometry with only one Cys-rich motif binding at any one time, with a resulting apparent K_D in the high nanomolar range. In other words, binding is relatively strong because of the presence of two binding sites for VPS29 on the same VARP molecule. Unfortunately, neither the K_D nor the stoichiometry of the binding of full-length proteins could be accurately determined, as all constructs containing both stacks of ankyrin repeats aggregated in ITC experiments, and all cocrystallization attempts have failed.

We therefore conclude that VARP is recruited on to endosomes through the direct interaction of its Cys-rich motifs with VPS29, thus placing its ability to bind and regulate the fusogenic potential of free VAMP7, and to interact with Rab32:GTP, on the surface of endosomes. In agreement with this model, GST pull-down experiments (Figure 5F) showed that VARP simultaneously binds to VPS29, Rab32/38, and VAMP7 in vitro.

A VARP/Retromer/VAMP7-Dependent Pathway Plays a Role in GLUT1 Traffic out of Endosomes

Recent studies have suggested that a number of cell surface receptors and transporters recycle from endosomes to the plasma membrane by a retromer-dependent pathway (Piotrowski et al., 2013; Steinberg et al., 2013; Temkin et al., 2011). In light of our results showing that VARP associates with retromer, we hypothesized that depleting VARP and/or VAMP7 (which cycles from endosomes/endolysosomes through the plasma membrane [Pryor et al., 2008] and binds directly to VARP) should also disrupt the trafficking of retromer-dependent cargoes. We chose to analyze the recently characterized retromer cargo GLUT1, which redistributes from a predominantly cell surface localization to an intracellular endosomal localization upon

Figure 5. VARP Binds to VPS29 via Two Conserved Zn^{2+} Coordinating Cys-Rich Motifs

(A) The two conserved Cys-rich motifs (conserved residues highlighted in pink with asterisks beneath them) in VARP from different species. Residue numbering is given for the human sequence.

(B) Proton-induced X-ray emission (PIXE) spectra for protein fragments containing the first (residues 396–460, left) and second (residues 692–746, right) Cys-rich motifs, with the Zn and Br peaks labeled above them. Black dots indicate the individual data points, and the red line is the fit.

(C–E) In (C), Y2H analysis is shown of the DNA binding domain (BD) fused to VPS29 and the activation domain (AD) fused to the indicated VARP fragments. Mutations within the Cys-rich motifs are labeled as follows in (C), (D), and (E): (first motif) H432A/L434A = HL1, C431S/C435S/C437S/C440S = 4C1; (second motif) H712A/L714A = HL2, C711S/C715S/C717S/C720S = 4C2. (D) Coomassie-stained gel of GST pull-down experiment showing interaction of VARP-His_{10} WT or the indicated VARP mutants with GST-VPS29 or with GST alone. (E) IF confocal microscopy of cytosol extracted HeLa cells stably expressing VARP-GFP WT or the indicated VARP mutants. GFP, green; VPS26, red; nuclei, blue, merged panels (MERGE). Colocalization coefficients of VPS26 versus GFP were as follows: WT, 0.9; HL1, 0.08; 4C1, 0.09; HL2, 0.07; 4C2, 0.1; HL1+HL2, 0; 4C1+ 4C2, 0. Scale bar, 20 μm.

(F) GST pull-down experiment showing the interaction of the indicated combinations of VARP-His_{10}, Rab32, and VAMP7 cytoplasmic domain (CD) (inputs, left three lanes), with either GST alone or GST-VPS29. The pull-down samples were western blotted for Rab32 and VAMP7.

See also Figure S5.

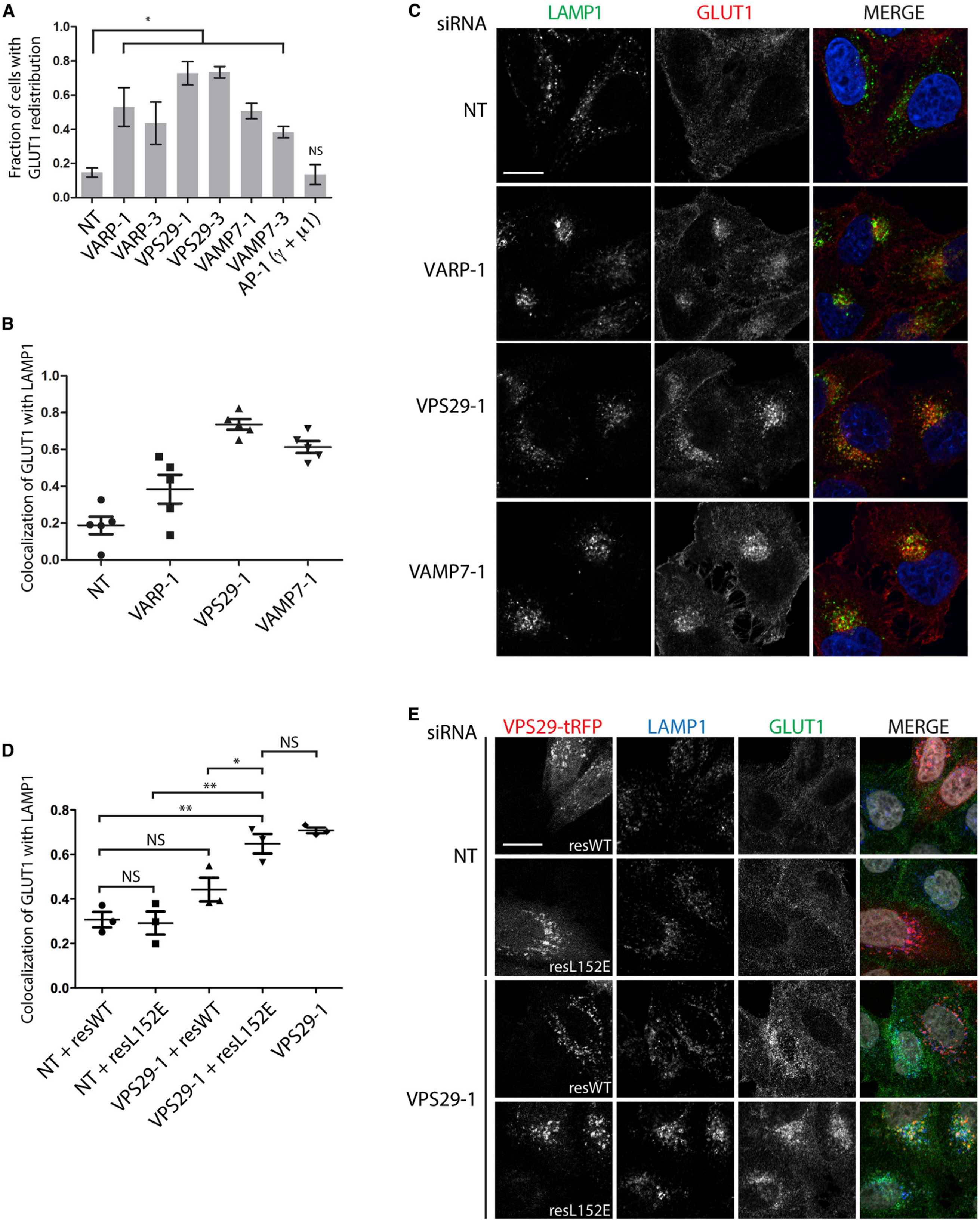

(legend on next page)

retromer depletion (Piotrowski et al., 2013; Steinberg et al., 2013). Consistent with these published findings, depletion of VPS29 in HeLa cells caused an increased intracellular distribution of GLUT1 as compared to control cells (Figure 6A; Figure S6A). A similar redistribution of GLUT1 was also observed upon knockdown of VARP and VAMP7, whereas knockdown of the clathrin adaptor AP-1 had no effect on GLUT1 distribution. We followed up on these initial observations of GLUT1 redistribution by measuring the colocalization of GLUT1 with the late endosomal/lysosomal membrane protein LAMP1, because Steinberg et al. (2013) had previously shown that depletion of the retromer subunit VPS35 increased such colocalization. We observed a marked increase in the colocalization of GLUT1 with LAMP1 after knockdown of VPS29, VARP, or VAMP7 (Figures 6B and 6C; Figures S6B and S6C) but, interestingly, not after depletion of the RabGAP TBC1D5 (Figures S6B and S6D) that binds to VPS29 at the same site as VARP (Harbour et al., 2010). Subsequent experiments with transiently transfected HeLa cells showed that siRNA-resistant WT VPS29-TagRFP, but not the L152E mutant that does not bind VARP, was able to rescue the increase in colocalization of GLUT1 with LAMP1 observed after knockdown of endogenous VPS29 (Figures 6D, 6E, and S6E). There was no statistically significant difference in colocalization of GLUT1 with LAMP1 after knockdown of VPS29 in cells expressing the siRNA-resistant L152E mutant when compared with nearby untransfected cells (Figures 6D and S6E). These data confirmed that the interaction between VARP and VPS29 was necessary to rescue the GLUT1/LAMP1 colocalization phenotype observed after knockdown of VPS29.

Taken together, our data demonstrate that VARP and VAMP7 act within the retromer-dependent recycling pathway that traffics the glucose transporter GLUT1 from endosomes to the plasma membrane. Our rescue experiments show that the VPS29 requirement for this pathway is dependent on its binding to VARP. Given the role of retromer in mediating traffic out of endosomes, and the knowledge that VARP binds the retromer subunit VPS29 and VAMP7 at different binding sites, we decided to study further whether the intracellular distribution of VAMP7 is affected by its association with VARP/retromer. Stable HeLa cell lines expressing WT VAMP7-HA or the D69A, E71F, S73D (DES) mutant VAMP7-HA, which we have previously shown cannot bind VARP (Schäfer et al., 2012, Kent et al., 2012), were examined by IF confocal microscopy. The DES VAMP7-HA mutant was primarily distributed to the perinuclear region as compared to WT VAMP7-HA, which had a more peripheral endosomal localization in addition to a perinuclear pool (Figure 7A). This difference in localization was quantified by measuring the degree of colocalization between VAMP7-HA and the trans-Golgi network (TGN) marker TGN46, with DES VAMP7-HA exhibiting a higher degree of colocalization (Figure 7B). The increased colocalization of DES VAMP7-HA with TGN46 was lost on treatment of the cells with chloroquine (CQ), which inhibits the trafficking of proteins from endosomes to the TGN (Chapman and Munro, 1994; Kent et al., 2012; Reaves and Banting, 1994) but only weakly affects the distribution of TGN46 (Ponnambalam et al., 1996), unlike the more marked effect on its ortholog TGN38 in rodent cells (Chapman and Munro, 1994). Our data with the DES mutant of VAMP7 imply that the normal cycling of VAMP7 within the endocytic sytem is dependent on its interaction with VARP, in addition to its interactions with Hrb (Pryor et al., 2008) and AP3-Δ-adaptin (Kent et al., 2012). We also observed an increase in colocalization of WT VAMP7-HA with TGN46 after knocking down the retromer subunits VPS29 or VPS35 (Figures 7C and 7D).

DISCUSSION

Retromer drives trafficking from endosomes/endolysosomes via tubular-vesicular carriers to either the Golgi or the cell surface (Cullen and Korswagen, 2012; Seaman, 2012; Seaman et al., 2013). Here, we have shown that VARP is recruited to endosomes through a direct interaction with retromer, where it participates in the retromer-dependent pathway that delivers the glucose transporter GLUT1 to the plasma membrane (Figures 7C and 7D).

The current working model for retromer function suggests that, in mammalian cells, the VPS26/35/29 subcomplex associates with endosomal membranes via Rab7A (Rojas et al., 2008; Seaman et al., 2009) and Snx3 (reviewed in Cullen and Korswagen, 2012; Seaman, 2012). Cargo selective functions have been suggested for VPS26 (Fjorback et al., 2012), VPS35 (Arighi et al., 2004; Nothwehr et al., 2000; Seaman, 2007), and the associated sorting nexin, Snx27 (Ghai et al., 2013; Steinberg et al., 2013; Temkin et al., 2011). Retromer also interacts with the WASH complex, which promotes localized actin polymerization to provide a structural and/or force-generating scaffold on which

Figure 6. The VARP/Retromer/VAMP7 Protein Network Is Required for GLUT1 Trafficking between Endosomes and the Cell Surface

(A) HeLa cells knocked down with single siRNA oligonucleotides at 100 nM (NT, nontargeting control). AP-1 γ and μ1 subunits were knocked down simultaneously (pool of four individual oligonucleotides at 20 nM for each subunit). Cells were imaged by IF confocal microscopy, and the fraction of cells with GLUT1 redistributed from the cell surface to an intracellular endosomal localization with the indicated knockdown was graphed (mean ± SEM of three independent experiments with >400 cells scored for each condition, $^*p < 0.05$).

(B and C) HeLa cells were knocked down as shown in (A), and cells were imaged by IF confocal microscopy. LAMP1, green; GLUT1, red; nuclei, blue, merged panels (MERGE). Five independent fields of cells were imaged for each condition, and the colocalization coefficient measuring the colocalization of GLUT1 with LAMP1 was obtained for each field. The graph in (B) is a representative experiment (mean ± SEM of five fields), with representative IF confocal images shown in (C).

(D and E) HeLa cells were knocked down as in (A) with either nontargeting control (NT) or VPS29-1 oligonucleotides. Twenty-four hours prior to fixation, cells were transfected with siRNA-resistant VPS29-tRFP WT (resWT) or L152E mutant (resL152E). Cells were imaged by IF confocal microscopy. tRFP, red; LAMP1, blue; GLUT1, green; nuclei, white, merged panels (MERGE). Individual VPS29-tRFP-expressing cells ($\geq$17 cells per condition) were identified, and the colocalization coefficient measuring the colocalization of GLUT1 with LAMP1 was obtained for each cell. Colocalization coefficients from three independent experiments are shown in (D) (mean ± SEM, $^*p < 0.05$, $^{**}p < 0.01$, NS, not significant, using a two-tailed unpaired t test), and representative cell images are shown in (E). Cells analyzed in the VP29-1 knockdown-only condition were cells not transfected by VPS29-tRFP within VPS29-1 + resL152E experiments.

Scale bars, 20 μm. See also Figure S6.

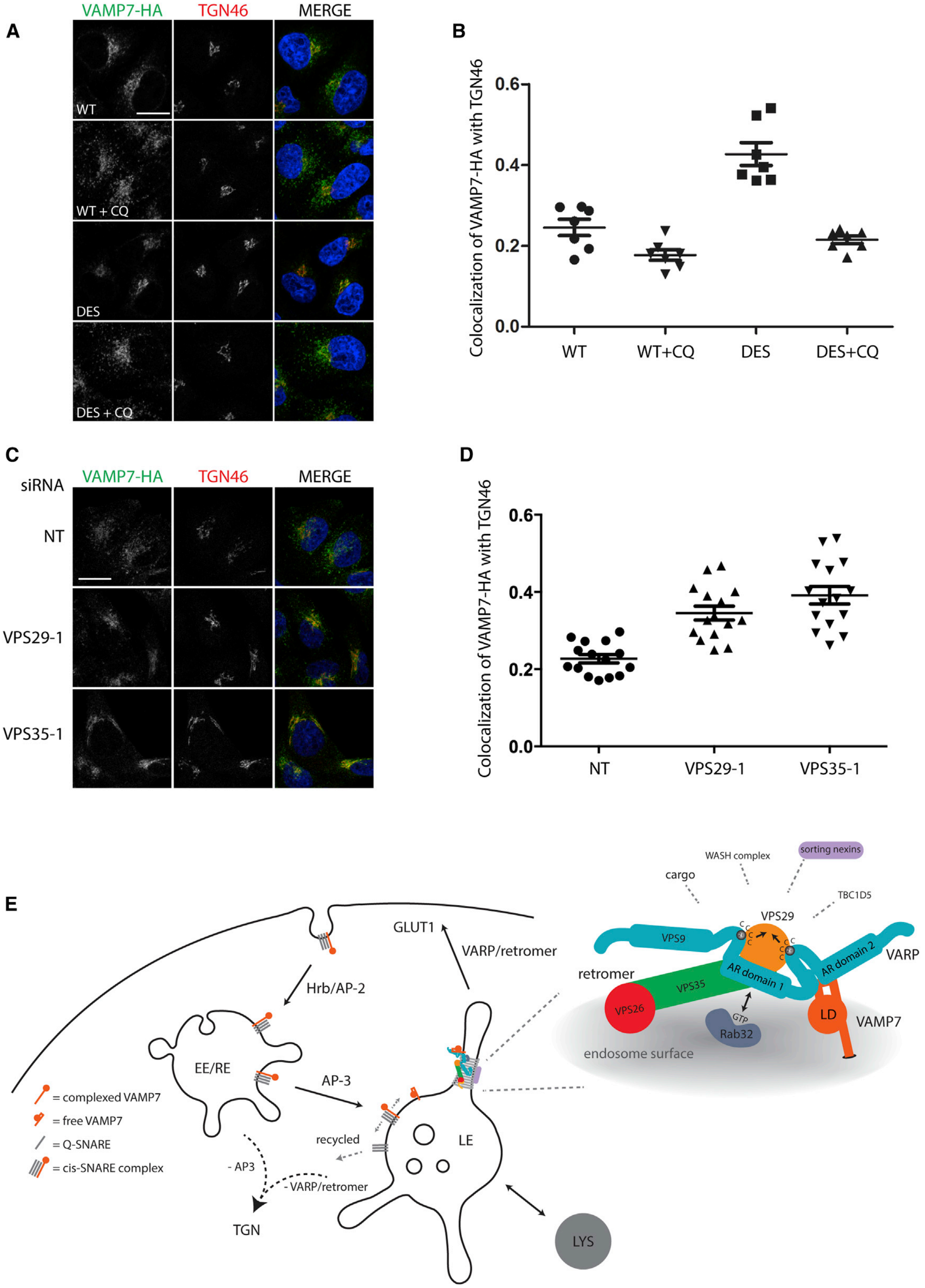

Figure 7. The Steady-State Localization of VAMP7 Depends on Its Interaction with VARP

(A and B) HeLa cells stably expressing VAMP7-HA WT or DES were imaged by IF confocal microscopy: HA, green; TGN46, red; nuclei, blue, merged panels (MERGE). Cells were either left untreated or treated with 100 μM CQ for 2 hr prior to fixation. Seven independent fields (≥ 10 cells per field) were imaged for each condition, and the colocalization coefficient measuring the colocalization of VAMP7-HA with TGN46 was obtained for each field [mean ± SEM in (B) with representative cell images in (A)].

(legend continued on next page)

endosomal tubular carriers may be formed (Derivery et al., 2009; Gomez and Billadeau, 2009; Harbour et al., 2010; Jia et al., 2012). The known destinations of retromer-derived carriers leaving endosomes are the Golgi and the cell surface (Cullen and Korswagen, 2012; Fjorback et al., 2012; Seaman, 2007, 2012; Steinberg et al., 2013; Temkin et al., 2011). The interaction between retromer and VARP described here places VARP firmly in the trafficking pathway from endosomes to the cell surface and further cements the role of retromer as a hub for recruiting key machinery required for trafficking out of endosomes (Harbour et al., 2010) (Figure 7C). The binding of VARP by retromer is mediated by the VPS29 subunit and involves the same cluster of hydrophobic residues that mediates the association of retromer with the RabGAP TBC1D5. It has been proposed that binding of TBC1D5 by retromer leads to retromer dissociation from membranes through increasing GTP hydrolysis of Rab7A, a Rab key to retromer recruitment (Harbour et al., 2010; Seaman et al., 2009) VARP association with retromer may inhibit the binding of TBC1D5, leading to a more persistent association of retromer with the membrane, thereby facilitating the sorting of cargo proteins into the endosome-to-cell surface pathway. Retromer was first identified in yeast (Seaman et al., 1997, 1998) and was shown to mediate endosome-to-Golgi retrieval. To date, there is no compelling evidence that retromer in yeast can drive endosome-to-cell surface traffic, and VARP is not present in yeast, consistent with the hypothesis that VARP regulates the activity of retromer in the endosome-to-cell surface pathway in mammalian cells (Gerondopoulos et al., 2012; Kloer et al., 2010; Nottingham et al., 2011; Rojas et al., 2008; Seaman et al., 2009; Stenmark, 2009). The reason for the VARP:Rab32 interaction remains unclear. As it is not required for VARP recruitment to endosomal membranes, we did not pursue it further in this study. However, our data are consistent with a model in which VARP is initially recruited to the endosome through its interaction with retromer. Subsequently, the VARP:retromer complex may either be partitioned into or contribute to the formation of a Rab32-defined endosomal subdomain, from which trafficking out of the endosome may occur.

VAMP7 mediates fusion of both endosomes and endolysosomes with lysosomes and fusion of endolysosomes and their derived carriers with the plasma membrane (reviewed in Chaineau et al., 2009). VAMP7 is then returned to its steady-state endolysosomal location through interactions with Hrb and AP-3 (Boucrot and Kirchhausen, 2007; Chaineau et al., 2008; Kent et al., 2012; Martinez-Arca et al., 2003; Pryor et al., 2004, 2008; Rao et al., 2004). The demonstration that VARP can interact simultaneously with retromer and VAMP7 suggests other potential reasons why the VARP:retromer interaction may be important in trafficking out of endosomes. While Hrb and AP-3 appear to be the major factors determining the endosomal localization of VAMP7 (see Kent et al., 2012), a role for VARP in VAMP7 distribution within the endosomal compartment is supported by our observation that a VARP binding-deficient mutant of VAMP7 is mislocalized away from endosomes in a similar manner to that observed in retromer-knockdown cells. The redistribution of VAMP7 to a TGN localization after loss of VARP interaction suggests that VARP:retromer is necessary to direct VAMP7 away from a retromer-independent endosome-to-Golgi retrieval pathway such as that used by furin (Chia et al., 2011). Alternatively, VAMP7 redistribution to the TGN could be caused, at least in part, by VARP operating at the TGN to sort VAMP7 into vesicles for transport to endosomes (Burgo et al., 2012). However, this is difficult to reconcile with the inability to detect obvious localization of WT VARP at the TGN in nonneuronal cells by IF (Zhang et al., 2006) or immunoelectron microscopy (Schäfer et al., 2012).

The incorporation of VAMP7 into retromer-dependent endosome-derived carriers could facilitate the subsequent fusion of these carriers with their final destinations (see also Burgo et al., 2012), consistent with our observation that both VAMP7 and retromer are required for delivery of the glucose transporter GLUT1 to the plasma membrane. In addition, once VARP is concentrated on an endosomal membrane by retromer, VARP has the potential to regulate the fusogenic ability of VAMP7 (Schäfer et al., 2012). Therefore, it is possible that control of endosome/lysosome fusion could, in part, be regulated by the amount of retromer on the endosome surface through its ability to recruit VARP.

In summary, through its multiple interactions, VARP can link the retromer complex (VPS26/35/29) and its associated proteins (including sorting nexins and the WASH complex) to both a key ancestral R-SNARE protein (VAMP7) (Filippini et al., 2001) involved in many membrane fusion events (Chaineau et al., 2009) and to an endosome-localized Rab (Figure 7). Thus, we propose that VARP is a key component of a protein network that both drives and controls traffic out of endosomes.

EXPERIMENTAL PROCEDURES

Protein Expression and Purification

Protein expression was performed in *E. coli* Rosetta 2 pLysS. Cells were grown at 37°C, and expression was induced by the addition of 500 μM IPTG. VARP expression was carried out at 16°C for 20–24 hr, while all other proteins were expressed at 22°C for 14–18 hr. Cells were lysed in 20 mM HEPES, pH 7.4, and 500 mM NaCl (buffer A), supplemented with 5 mM $MgCl_2$, 1 mM $MnCl_2$, 5 mM dithiothreitol (DTT), 4 μg/ml DNase, and protease inhibitors and AEBSF. Seleno-methionine proteins were expressed in *E. coli* B834 using M9 media supplemented as in Collins et al. (2008).

Protein Purification and VARP:Rab Complex Reconstitution

Proteins were first purified on GSH-sepharose, and the GST tag was cleaved off by incubating the beads with PreScission Protease (GE Healthcare)

(C) IF confocal microscopy of VAMP7-HA HeLa cells knocked down with the indicated siRNA oligonucleotides at 100 nM (NT, nontargeting control, VPS29-1, VPS35-1): HA, green; TGN46, red; nuclei, blue, merged panels. Scale bars, 20 μm.

(D) Fifteen independent fields (≥ 10 cells per field) were imaged for each condition, and the colocalization coefficient measuring the colocalization of VAMP7-HA with TGN46 was obtained for each field (mean ± SEM). Data shown are representative of three independent experiments. NT, nontargeting control.

(E) Model of the VARP/retromer/VAMP7 protein network.

VAMP7 is endocytosed from the plasma membrane as a cis-SNARE complex through the action of Hrb/AP-2 and is subsequently delivered to a late endosomal compartment through the action of AP-3. VARP is recruited to endosomal membranes by the retromer complex through its direct interaction with VPS29, thus allowing VARP to bind to free VAMP7 on the surface of endosomes.

overnight at 4°C. His-tagged $VARP_{451–640}His_6$ was further purified on Ni-NTA agarose. All proteins were purified by gel filtration on Superdex 75 in buffer A supplemented with 10 mM DTT and additionally with 5 mM $MgCl_2$ and 10 μM GppCp or GTPγS in the case of Rab32/38. Complexes were formed by mixing VARP with a GTPase in a molar excess of 1:1.25 for 1 hr at 4°C and finally subjecting them to gel filtration on Superdex 75 equilibrated in buffer A containing 5 mM $MgCl_2$, 10 μM GppCp or GTPγS, and 10 mM DTT. Fractions of 1:1 complex as assessed by SDS-PAGE were pooled and concentrated for crystallization. Proteins for ITC were gel filtered on Superdex 75 in 50 mM HEPES, pH 7.4, 200 mM NaCl, 5 mM $MgCl_2$, 1 mM TCEP.

Protein Purification for Pull-Downs

$VARP_{1–1050}His_{10}$ WT and mutants were purified in 500 mM NaCl, 20mM Tris pH8.5, (buffer B) as in (Schäfer et al., 2012). GST-tagged proteins were purified on GSH-sepharose and eluted in buffer B containing 30 mM reduced glutathione, or the GST was removed by overnight incubation with PreScission Protease or thrombin as appropriate. Proteins were finally purified by gel filtration on Superdex 200 in buffer B that, in the case of Rabs, was supplemented with 5 mM $MgCl_2$ and 10 μM of matching nucleotide.

Crystallization and Structure Determination

Crystals of $VARP_{451–640}His_6$: $Rab32_{1–225}$(Q85L) were grown by sitting drop vapor diffusion at 18°C against well solutions containing 14%–19% (w/v) of polyethylene glycol 3350 and 200 mM sodium citrate, pH 8.0, at an initial complex concentration of 2.5 mg/ml. Cryoprotected crystals exhibited anisotropic diffraction at 100 K and diffracted in the best directions to 2.8 Å. The structure was solved by molecular replacement with Phaser (McCoy et al., 2007), using models based on the structures of Rab7 (Protein Data Bank [PDB] ID 1YHN) and the VARP-ANKR domain 2 structure (PDB ID 4B93). Crystals of VARP-ANKR domain 1:SeMet- $Rab32_{1–225}$(Q85L/V100M/Q153M/V158M/I192M) and of VARP-ANKR domain 1:SeMet-Rab38 (Q69L) diffracted in their best directions to 3 Å and 4.5 Å, respectively. Their structures were solved by molecular replacement using the coordinates of native VARP-ANKR domain 1:Rab32Q85L complex as the search model. For a full description of structure determination, see Supplemental Information.

Protein:Protein Interaction Studies

Y2H for screening and analyzing specific interactions was performed using pGBT9 and pGAD-C vectors and the HF7c reporter strain as previously described (Harbour et al., 2010; Kent et al., 2012; Pryor et al., 2008). Growth on plates lacking histidine indicates that interaction occurred. For GST pull-down assays, 25–50 μg of the GST bait protein was incubated with 300 μg prey protein and 30–60 μl of a 50% (v/v) GSH-sepharose slurry in a total volume of 500 μl (5 mM $MgCl_2$, 0.5mM TCEP, and 100 μM of the matching nucleotide was included if a Rab was present and 0.5mM $ZnCl_2$ if a Cys-rich motif was present). The reactions were incubated for 1 hr at 4°C, and the beads were washed four times with 1 ml of buffer supplemented with 1% (w/v) NP40. Proteins were eluted from the beads using buffer containing 50 mM reduced GSH. Samples were analyzed on SDS-PAGE and/or western blots, which were probed with HisProbe-horseradish peroxidase conjugate or appropriate antibodies.

Isothermal Titration Calorimetry

Experiments were performed using a Nano ITC from TA Instruments. VARP constructs at 60 μM were placed in the cell at 4°C, and Rab constructs at 300 μM were titrated with 24 injections of 2 μl with injections separated by 5 min. A minimum of three independent runs that showed clear saturation of binding were used to calculate the mean K_D of the binding reaction, its stoichiometry, and their corresponding SD values. Analysis of results and final figures were carried out using the NanoAnalyze Software.

microPIXE

Samples were gel filtered at 10 mg/ml into 20 mM Tris, pH 7.4, 100 mM NaBr, and 0.5 mM TCEP to ensure low chlorine content and no nonprotein sulfur. The resulting spectra of X-rays were sorted into two-dimensional elemental maps from which the stoichiometric ratios of sulfur to other elements of interest were obtained.

Immunoprecipitation and Mass Spectrometry

HeLaM cells stably expressing VPS29-EGFP or EGFP-VPS35 were lysed using PBS containing 1% Triton X-100. Following lysis, the samples were cleared by centrifugation at 10,000 × g for 5 min. The supernatant was transferred to a fresh tube and treated with protein-A sepharose (50 μl of a 25% slurry) for 30 min at 4°C on a rotating wheel. The lysates were then cleared again by centrifugation before being treated with a polyclonal anti-GFP antibody followed by protein-A sepharose essentially as described in Harbour et al. (2010). Following washes, proteins bound to protein-A sepharose were eluted using 200 mM glycine-HCl, pH 2.3, and then precipitated with ice-cold acetone. The precipitated proteins were digested with trypsin in solution and analyzed with an LTQ-OrbiTrap XL (Thermo) mass spectrometer essentially as described elsewhere (Weekes et al., 2013). For mass spectrometric analysis, four dishes (140 mm in diameter) of cells were used for each cell line. For western blotting analysis, a single dish of cells was used for each cell line, and the washed protein-A sepharose was dessicated in a SpeedVac prior to resuspension in SDS-PAGE sample buffer omitting the glycine elution and acetone precipitation steps.

Mammalian Cell Culture, Microscopy, and Western Blotting

HeLaM cell populations stably expressing VARP-GFP constructs were generated using the pLXIN retroviral system (Clontech). When indicated, HeLaM cytosol was extracted prior to fixation by rinsing cells briefly with PBS, followed by incubation for 1 min in PBS (with Ca^{2+} and Mg^{2+}) containing 0.05% saponin, and then immediately formaldehyde fixed. IF confocal microscopy and western blotting were carried out essentially as described elsewhere (Schäfer et al., 2012). For quantitative IF confocal microscopy of GLUT1 and LAMP1 colocalization, cell fields (three independent experiments with five fields each per condition, average of ≥8 cells per field) were randomly selected based on nuclear stain and focused using the LAMP1 signal. Single confocal images were acquired corresponding to 1 Airy unit, and the degree of colocalization of two channels was measured by Manders M_1/M_2 colocalization coefficients (Manders et al., 1993) using Zen software (Carl Zeiss). To negate the effects of inherent cell-to-cell variability of GLUT1 surface staining, the fraction of LAMP1 signal colocalizing with GLUT1 was measured for each field.

ACCESSION NUMBERS

The PDB accession number for the structures presented here is 4CYM, and for structure factors, it is r4cymsf.

SUPPLEMENTAL INFORMATION

Supplemental Information includes Supplemental Experimental Procedures, six figures, and two tables and can be found with this article online at http://dx.doi.org/10.1016/j.devcel.2014.04.010.

AUTHOR CONTRIBUTIONS

I.P.-D., I.B.S., G.G.H., A.J.M., and P.R.E. carried out work on the Rab32:VARP complex. G.G.H., L.P.J., L.W., I.B.S., S.R.G., M.E.H., M.N.J.S., and D.J.O. carried out work on VARP:Retromer. O.B.Z. and E.F.G. carried out the microPIXE analysis. J.P.L., M.N.J.S., and D.J.O. conceived and initiated the study. All authors contributed to the design of the study and the writing of the manuscript.

ACKNOWLEDGMENTS

We thank the beamline staff at IO3 SLS Diamond plc; Robin Antrobus for mass spectrometry analysis; Andrew Peden, Miguel Seabra, Scottie Robinson, and Sean Munro for reagents; and Geoff Grime at the Surrey Ion Beam Centre for assistance. G.G.H. was funded by a postdoctoral fellowship from the Canadian Institutes of Health Research; I.P.-D. was funded by a Federation of European Biochemical Societies Long-Term Fellowship; and L.W. was funded by an EMBO Long-Term Fellowship. I.P.D., I.B.S., and P.R.E. were funded by Medical Research Council (MRC) grant U105178845; L.P.J. and

D.J.O. were funded by a Wellcome Trust fellowship; G.G.H., L.W., S.R.G., and J.P.L. were funded by an MRC programme grant (G0900113); O.B.Z. was funded by an Engineering and Physical Sciences Research Council-Doctoral Training Centre studentship; and M.E.H. and M.N.J.S. were funded by an MRC Senior Research Fellowship awarded to M.N.J.S. (G0701444). The Cambridge Institute for Medical Research is supported by a Wellcome Trust Strategic Award (100140).

Received: August 2, 2013
Revised: February 3, 2014
Accepted: April 9, 2014
Published: May 22, 2014

REFERENCES

Ambrosio, A.L., Boyle, J.A., and Di Pietro, S.M. (2012). Mechanism of platelet dense granule biogenesis: study of cargo transport and function of Rab32 and Rab38 in a model system. Blood *120*, 4072–4081.

Arighi, C.N., Hartnell, L.M., Aguilar, R.C., Haft, C.R., and Bonifacino, J.S. (2004). Role of the mammalian retromer in sorting of the cation-independent mannose 6-phosphate receptor. J. Cell Biol. *165*, 123–133.

Bal, M., Leitz, J., Reese, A.L., Ramirez, D.M., Durakoglugil, M., Herz, J., Monteggia, L.M., and Kavalali, E.T. (2013). Reelin mobilizes a VAMP7-dependent synaptic vesicle pool and selectively augments spontaneous neurotransmission. Neuron *80*, 934–946.

Boucrot, E., and Kirchhausen, T. (2007). Endosomal recycling controls plasma membrane area during mitosis. Proc. Natl. Acad. Sci. USA *104*, 7939–7944.

Bultema, J.J., Ambrosio, A.L., Burek, C.L., and Di Pietro, S.M. (2012). BLOC-2, AP-3, and AP-1 proteins function in concert with Rab38 and Rab32 proteins to mediate protein trafficking to lysosome-related organelles. J. Biol. Chem. *287*, 19550–19563.

Burgo, A., Sotirakis, E., Simmler, M.C., Verraes, A., Chamot, C., Simpson, J.C., Lanzetti, L., Proux-Gillardeaux, V., and Galli, T. (2009). Role of Varp, a Rab21 exchange factor and TI-VAMP/VAMP7 partner, in neurite growth. EMBO Rep. *10*, 1117–1124.

Burgo, A., Proux-Gillardeaux, V., Sotirakis, E., Bun, P., Casano, A., Verraes, A., Liem, R.K., Formstecher, E., Coppey-Moisan, M., and Galli, T. (2012). A molecular network for the transport of the TI-VAMP/VAMP7 vesicles from cell center to periphery. Dev. Cell *23*, 166–180.

Chaineau, M., Danglot, L., Proux-Gillardeaux, V., and Galli, T. (2008). Role of HRB in clathrin-dependent endocytosis. J. Biol. Chem. *283*, 34365–34373.

Chaineau, M., Danglot, L., and Galli, T. (2009). Multiple roles of the vesicular-SNARE TI-VAMP in post-Golgi and endosomal trafficking. FEBS Lett. *583*, 3817–3826.

Chapman, R.E., and Munro, S. (1994). Retrieval of TGN proteins from the cell surface requires endosomal acidification. EMBO J. *13*, 2305–2312.

Chia, P.Z., Gasnereau, I., Lieu, Z.Z., and Gleeson, P.A. (2011). Rab9-dependent retrograde transport and endosomal sorting of the endopeptidase furin. J. Cell Sci. *124*, 2401–2413.

Cohen-Solal, K.A., Sood, R., Marin, Y., Crespo-Carbone, S.M., Sinsimer, D., Martino, J.J., Robbins, C., Makalowska, I., Trent, J., and Chen, S. (2003). Identification and characterization of mouse Rab32 by mRNA and protein expression analysis. Biochim. Biophys. Acta *1651*, 68–75.

Collins, B.M., Skinner, C.F., Watson, P.J., Seaman, M.N., and Owen, D.J. (2005). Vps29 has a phosphoesterase fold that acts as a protein interaction scaffold for retromer assembly. Nat. Struct. Mol. Biol. *12*, 594–602.

Collins, B.M., Norwood, S.J., Kerr, M.C., Mahony, D., Seaman, M.N., Teasdale, R.D., and Owen, D.J. (2008). Structure of Vps26B and mapping of its interaction with the retromer protein complex. Traffic *9*, 366–379.

Cullen, P.J., and Korswagen, H.C. (2012). Sorting nexins provide diversity for retromer-dependent trafficking events. Nat. Cell Biol. *14*, 29–37.

Derivery, E., Sousa, C., Gautier, J.J., Lombard, B., Loew, D., and Gautreau, A. (2009). The Arp2/3 activator WASH controls the fission of endosomes through a large multiprotein complex. Dev. Cell *17*, 712–723.

Fader, C.M., Sánchez, D.G., Mestre, M.B., and Colombo, M.I. (2009). TI-VAMP/VAMP7 and VAMP3/cellubrevin: two v-SNARE proteins involved in specific steps of the autophagy/multivesicular body pathways. Biochim. Biophys. Acta *1793*, 1901–1916.

Filippini, F., Rossi, V., Galli, T., Budillon, A., D'Urso, M., and D'Esposito, M. (2001). Longins: a new evolutionary conserved VAMP family sharing a novel SNARE domain. Trends Biochem. Sci. *26*, 407–409.

Fjorback, A.W., Seaman, M., Gustafsen, C., Mehmedbasic, A., Gokool, S., Wu, C., Militz, D., Schmidt, V., Madsen, P., Nyengaard, J.R., et al. (2012). Retromer binds the FANSHY sorting motif in SorLA to regulate amyloid precursor protein sorting and processing. J. Neurosci. *32*, 1467–1480.

Garman, E.F., and Grime, G.W. (2005). Elemental analysis of proteins by microPIXE. Prog. Biophys. Mol. Biol. *89*, 173–205.

Gerondopoulos, A., Langemeyer, L., Liang, J.R., Linford, A., and Barr, F.A. (2012). BLOC-3 mutated in Hermansky-Pudlak syndrome is a Rab32/38 guanine nucleotide exchange factor. Curr. Biol. *22*, 2135–2139.

Ghai, R., Bugarcic, A., Liu, H., Norwood, S.J., Skeldal, S., Coulson, E.J., Li, S.S., Teasdale, R.D., and Collins, B.M. (2013). Structural basis for endosomal trafficking of diverse transmembrane cargos by PX-FERM proteins. Proc. Natl. Acad. Sci. USA *110*, E643–E652.

Gomez, T.S., and Billadeau, D.D. (2009). A FAM21-containing WASH complex regulates retromer-dependent sorting. Dev. Cell *17*, 699–711.

Harbour, M.E., Breusegem, S.Y., Antrobus, R., Freeman, C., Reid, E., and Seaman, M.N. (2010). The cargo-selective retromer complex is a recruiting hub for protein complexes that regulate endosomal tubule dynamics. J. Cell Sci. *123*, 3703–3717.

Harbour, M.E., Breusegem, S.Y., and Seaman, M.N. (2012). Recruitment of the endosomal WASH complex is mediated by the extended 'tail' of Fam21 binding to the retromer protein Vps35. Biochem. J. *442*, 209–220.

Helfer, E., Harbour, M.E., Henriot, V., Lakisic, G., Sousa-Blin, C., Volceanov, L., Seaman, M.N., and Gautreau, A. (2013). Endosomal recruitment of the WASH complex: active sequences and mutations impairing interaction with the retromer. Biol. Cell *105*, 191–207.

Jia, D., Gomez, T.S., Billadeau, D.D., and Rosen, M.K. (2012). Multiple repeat elements within the FAM21 tail link the WASH actin regulatory complex to the retromer. Mol. Biol. Cell *23*, 2352–2361.

Kent, H.M., Evans, P.R., Schäfer, I.B., Gray, S.R., Sanderson, C.M., Luzio, J.P., Peden, A.A., and Owen, D.J. (2012). Structural basis of the intracellular sorting of the SNARE VAMP7 by the AP3 adaptor complex. Dev. Cell *22*, 979–988.

Kloer, D.P., Rojas, R., Ivan, V., Moriyama, K., van Vlijmen, T., Murthy, N., Ghirlando, R., van der Sluijs, P., Hurley, J.H., and Bonifacino, J.S. (2010). Assembly of the biogenesis of lysosome-related organelles complex-3 (BLOC-3) and its interaction with Rab9. J. Biol. Chem. *285*, 7794–7804.

Klöpper, T.H., Kienle, N., Fasshauer, D., and Munro, S. (2012). Untangling the evolution of Rab G proteins: implications of a comprehensive genomic analysis. BMC Biol. *10*, 71.

Krissinel, E., and Henrick, K. (2007). Inference of macromolecular assemblies from crystalline state. J. Mol. Biol. *372*, 774–797.

Lee, M.T., Mishra, A., and Lambright, D.G. (2009). Structural mechanisms for regulation of membrane traffic by rab GTPases. Traffic *10*, 1377–1389.

Manders, E.M.M., Verbeek, F.J., and Aten, J.A. (1993). Measurement of co-localization of objects in dual-colour confocal images. J. Microsc. *169*, 375–382.

Martinez-Arca, S., Rudge, R., Vacca, M., Raposo, G., Camonis, J., Proux-Gillardeaux, V., Daviet, L., Formstecher, E., Hamburger, A., Filippini, F., et al. (2003). A dual mechanism controlling the localization and function of exocytic v-SNAREs. Proc. Natl. Acad. Sci. USA *100*, 9011–9016.

McCoy, A.J., Grosse-Kunstleve, R.W., Adams, P.D., Winn, M.D., Storoni, L.C., and Read, R.J. (2007). Phaser crystallographic software. J. Appl. Cryst. *40*, 658–674.

Monteiro, P., Rossé, C., Castro-Castro, A., Irondelle, M., Lagoutte, E., Paul-Gilloteaux, P., Desnos, C., Formstecher, E., Darchen, F., Perrais, D., et al.

(2013). Endosomal WASH and exocyst complexes control exocytosis of MT1-MMP at invadopodia. J. Cell Biol. *203*, 1063–1079.

Moreau, K., Ravikumar, B., Renna, M., Puri, C., and Rubinsztein, D.C. (2011). Autophagosome precursor maturation requires homotypic fusion. Cell *146*, 303–317.

Nothwehr, S.F., Ha, S.A., and Bruinsma, P. (2000). Sorting of yeast membrane proteins into an endosome-to-Golgi pathway involves direct interaction of their cytosolic domains with Vps35p. J. Cell Biol. *151*, 297–310.

Nottingham, R.M., Ganley, I.G., Barr, F.A., Lambright, D.G., and Pfeffer, S.R. (2011). RUTBC1 protein, a Rab9A effector that activates GTP hydrolysis by Rab32 and Rab33B proteins. J. Biol. Chem. *286*, 33213–33222.

Osanai, K., Takahashi, K., Nakamura, K., Takahashi, M., Ishigaki, M., Sakuma, T., Toga, H., Suzuki, T., and Voelker, D.R. (2005). Expression and characterization of Rab38, a new member of the Rab small G protein family. Biol. Chem. *386*, 143–153.

Panic, B., Perisic, O., Veprintsev, D.B., Williams, R.L., and Munro, S. (2003). Structural basis for Arl1-dependent targeting of homodimeric GRIP domains to the Golgi apparatus. Mol. Cell *12*, 863–874.

Park, M., Serpinskaya, A.S., Papalopulu, N., and Gelfand, V.I. (2007). Rab32 regulates melanosome transport in Xenopus melanophores by protein kinase a recruitment. Curr. Biol. *17*, 2030–2034.

Piotrowski, J.T., Gomez, T.S., Schoon, R.A., Mangalam, A.K., and Billadeau, D.D. (2013). WASH knockout T cells demonstrate defective receptor trafficking, proliferation, and effector function. Mol. Cell. Biol. *33*, 958–973.

Ponnambalam, S., Girotti, M., Yaspo, M.L., Owen, C.E., Perry, A.C., Suganuma, T., Nilsson, T., Fried, M., Banting, G., and Warren, G. (1996). Primate homologues of rat TGN38: primary structure, expression and functional implications. J. Cell Sci. *109*, 675–685.

Pryor, P.R., Mullock, B.M., Bright, N.A., Lindsay, M.R., Gray, S.R., Richardson, S.C., Stewart, A., James, D.E., Piper, R.C., and Luzio, J.P. (2004). Combinatorial SNARE complexes with VAMP7 or VAMP8 define different late endocytic fusion events. EMBO Rep. *5*, 590–595.

Pryor, P.R., Jackson, L., Gray, S.R., Edeling, M.A., Thompson, A., Sanderson, C.M., Evans, P.R., Owen, D.J., and Luzio, J.P. (2008). Molecular basis for the sorting of the SNARE VAMP7 into endocytic clathrin-coated vesicles by the ArfGAP Hrb. Cell *134*, 817–827.

Rao, S.K., Huynh, C., Proux-Gillardeaux, V., Galli, T., and Andrews, N.W. (2004). Identification of SNAREs involved in synaptotagmin VII-regulated lysosomal exocytosis. J. Biol. Chem. *279*, 20471–20479.

Reaves, B., and Banting, G. (1994). Vacuolar ATPase inactivation blocks recycling to the trans-Golgi network from the plasma membrane. FEBS Lett. *345*, 61–66.

Rojas, R., van Vlijmen, T., Mardones, G.A., Prabhu, Y., Rojas, A.L., Mohammed, S., Heck, A.J., Raposo, G., van der Sluijs, P., and Bonifacino, J.S. (2008). Regulation of retromer recruitment to endosomes by sequential action of Rab5 and Rab7. J. Cell Biol. *183*, 513–526.

Schäfer, I.B., Hesketh, G.G., Bright, N.A., Gray, S.R., Pryor, P.R., Evans, P.R., Luzio, J.P., and Owen, D.J. (2012). The binding of Varp to VAMP7 traps VAMP7 in a closed, fusogenically inactive conformation. Nat. Struct. Mol. Biol. *19*, 1300–1309.

Seaman, M.N. (2007). Identification of a novel conserved sorting motif required for retromer-mediated endosome-to-TGN retrieval. J. Cell Sci. *120*, 2378–2389.

Seaman, M.N. (2012). The retromer complex - endosomal protein recycling and beyond. J. Cell Sci. *125*, 4693–4702.

Seaman, M.N.J., Marcusson, E.G., Cereghino, J.-L., and Emr, S.D. (1997). Endosome to Golgi retrieval of the vacuolar protein sorting receptor, Vps10p, requires the function of the VPS29, VPS30, and VPS35 gene products. J. Cell Biol. *137*, 79–92.

Seaman, M.N.J., McCaffrey, J.M., and Emr, S.D. (1998). A membrane coat complex essential for endosome to Golgi retrograde transport in yeast. J. Cell Biol. *142*, 665–681.

Seaman, M.N., Harbour, M.E., Tattersall, D., Read, E., and Bright, N. (2009). Membrane recruitment of the cargo-selective retromer subcomplex is catalysed by the small GTPase Rab7 and inhibited by the Rab-GAP TBC1D5. J. Cell Sci. *122*, 2371–2382.

Seaman, M.N., Gautreau, A., and Billadeau, D.D. (2013). Retromer-mediated endosomal protein sorting: all WASHed up! Trends Cell Biol. *23*, 522–528.

Steinberg, F., Gallon, M., Winfield, M., Thomas, E.C., Bell, A.J., Heesom, K.J., Tavaré, J.M., and Cullen, P.J. (2013). A global analysis of SNX27-retromer assembly and cargo specificity reveals a function in glucose and metal ion transport. Nat. Cell Biol. *15*, 461–471.

Stenmark, H. (2009). Rab GTPases as coordinators of vesicle traffic. Nat. Rev. Mol. Cell Biol. *10*, 513–525.

Swarbrick, J.D., Shaw, D.J., Chhabra, S., Ghai, R., Valkov, E., Norwood, S.J., Seaman, M.N., and Collins, B.M. (2011). VPS29 is not an active metallo-phosphatase but is a rigid scaffold required for retromer interaction with accessory proteins. PLoS ONE *6*, e20420.

Tamura, K., Ohbayashi, N., Maruta, Y., Kanno, E., Itoh, T., and Fukuda, M. (2009). Varp is a novel Rab32/38-binding protein that regulates Tyrp1 trafficking in melanocytes. Mol. Biol. Cell *20*, 2900–2908.

Tamura, K., Ohbayashi, N., Ishibashi, K., and Fukuda, M. (2011). Structure-function analysis of VPS9-ankyrin-repeat protein (Varp) in the trafficking of tyrosinase-related protein 1 in melanocytes. J. Biol. Chem. *286*, 7507–7521.

Temkin, P., Lauffer, B., Jäger, S., Cimermancic, P., Krogan, N.J., and von Zastrow, M. (2011). SNX27 mediates retromer tubule entry and endosome-to-plasma membrane trafficking of signalling receptors. Nat. Cell Biol. *13*, 715–721.

Wang, F., Zhang, H., Zhang, X., Wang, Y., Ren, F., Zhang, X., Zhai, Y., and Chang, Z. (2008). Varp interacts with Rab38 and functions as its potential effector. Biochem. Biophys. Res. Commun. *372*, 162–167.

Wang, C., Liu, Z., and Huang, X. (2012). Rab32 is important for autophagy and lipid storage in Drosophila. PLoS ONE *7*, e32086.

Wasmeier, C., Romao, M., Plowright, L., Bennett, D.C., Raposo, G., and Seabra, M.C. (2006). Rab38 and Rab32 control post-Golgi trafficking of melanogenic enzymes. J. Cell Biol. *175*, 271–281.

Weekes, M.P., Tan, S.Y., Poole, E., Talbot, S., Antrobus, R., Smith, D.L., Montag, C., Gygi, S.P., Sinclair, J.H., and Lehner, P.J. (2013). Latency-associated degradation of the MRP1 drug transporter during latent human cytomegalovirus infection. Science *340*, 199–202.

Zhang, X., He, X., Fu, X.Y., and Chang, Z. (2006). Varp is a Rab21 guanine nucleotide exchange factor and regulates endosome dynamics. J. Cell Sci. *119*, 1053–1062.

Developmental Cell, Volume *29*

Supplemental Information

VARP Is Recruited onto Endosomes by Direct Interaction with Retromer, Where Together They Function in Export to the Cell Surface

Geoffrey G. Hesketh, Inmaculada Pérez-Dorado, Lauren P. Jackson, Lena Wartosch, Ingmar B. Schäfer, Sally R. Gray, Airlie J. McCoy, Oliver B. Zeldin, Elspeth F. Garman, Michael E. Harbour, Philip R. Evans, Matthew N.J. Seaman, J. Paul Luzio, and David J. Owen

Figure S1. Structure of the VARP ANKRD1:Rab32 complex
Related to Figure 1

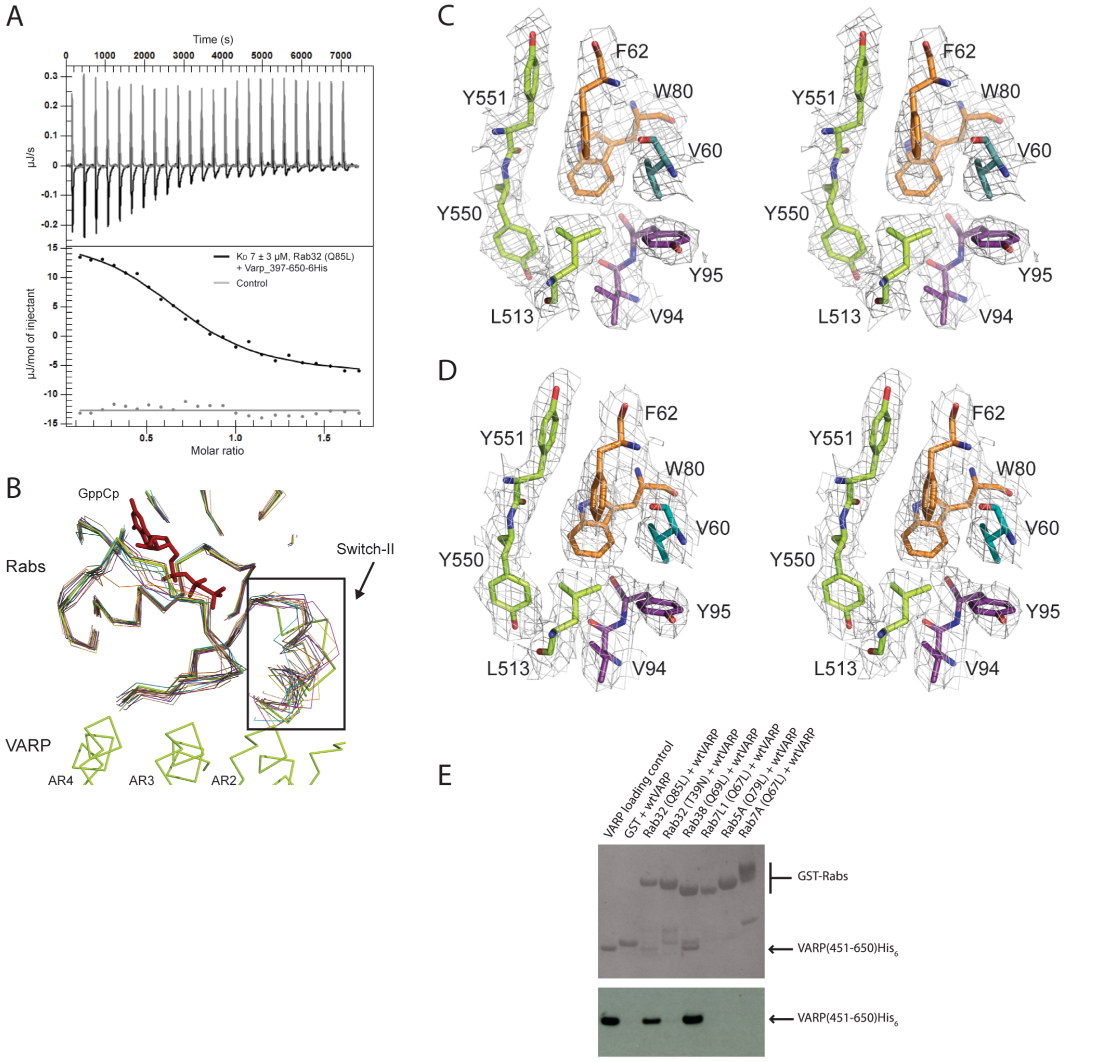

FIGURE S1

(A) Binding of VARP residues 397-650 to Rab32(Q85L) residues 1-225 analysed by ITC gave an estimated K_D of 7±3μM.

(B) Differences in the conformation of the GTPase in VARP ANKRD1:Rab32 complex compared to those in other Rab:effector complexes. Ribbon representation VARP:Rab32 (thicker and in green) superimposed with other Rab:GTPs: 1ZBD (yellow); 1TUE (red); 1Z0J (pink); 1Z0k (orange); 2D7C (light yellow); 2HV8 (magenta); 3BBP (purple); 2ZET (grey); 1YHN (cyan); 3BC1 (green-cyan); 3MJH (light blue); 3QBT (dark blue); 3TNF (brown); 3TKL (dark-green); and 3CWZ (beige). The Switch II region is highlighted by boxing. The only Rab effector shown is VARP.

(C) and (D), stereoviews showing a detail of the final refined 2mFo-DFc electron density maps contoured at 1.0 sigma for native (C) and SeMet-derivative VARP-(D) ANKRD1:Rab32 complexes.

(E) 'GST pull downs' using VARP451-640His$_6$ and GST tagged versions of the GTP locked forms of the endosomal Rabs, Rab5, Rab7, Rab7L1, Rab32 and Rab38 to show the specificity of the interaction for Rab32/38.

Figure S2 Analysis of the 'cross dimer' of the VARP-ANKRD1:Rab32 heterodimer.
Related to Figure 2

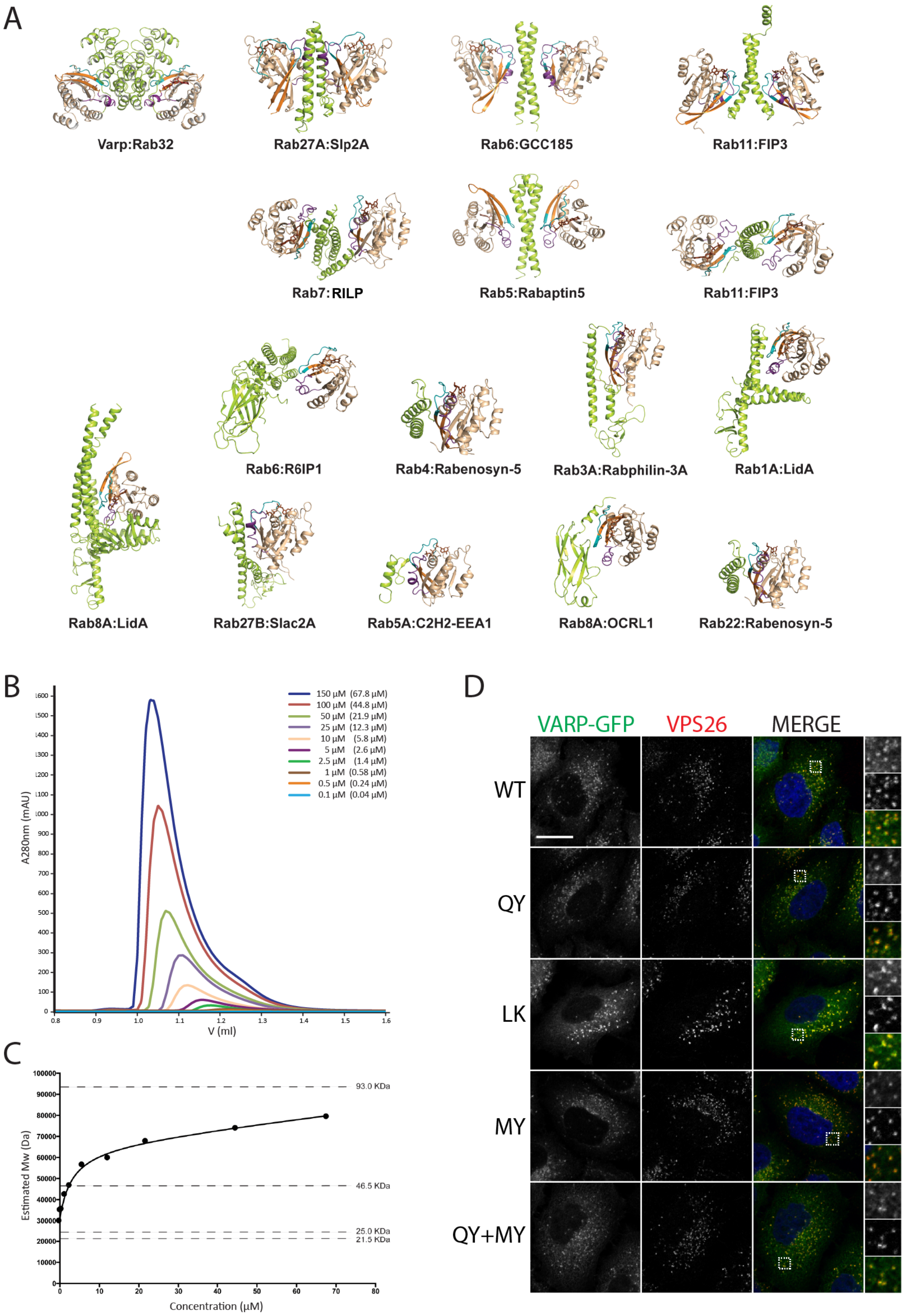

FIGURE S2

(A) Structures of various Rab:Rab effector complexes. VARP:Rab32 (4CYM); Rab27A:Slp2A (3BC1); Rab6:GCC185 (3BBP); Rab11:FIP3 (2HV8); Rab7:RILP (1YHN); Rab5:Rabaptin5 (1TUE); Rab11:FIP3 (2D7C); Rab6:R6IP1 (3CWZ);

Rab3A:Rabphilin-3A (1ZBD); Rab1A:LidA (3TKL); Rab8A:LidA (3TNF); Rab27B:Slac2A (2ZET); Rab5A:C2H2-EEA1 (3MJH); Rab8:OCRL1 (3QBT); Rab4:Rabenosyn-5 (1Z0K); and Rab22:Rabenosyn-5 (1Z0J). The color code throughout is green for the Rab-effectors, and beige for the Rabs with Switch-I, inter-switch, and Switch-II regions highlighted in cyan, orange, and purple respectively.

(B) and (C) Analytical gel filtration of VARP ANKRD1:Rab32(Q85L) complex. Samples of the complex were loaded at ten different concentrations ranging from 0.1 μM to 150 μM. In each case, calculated concentrations in the peak are indicated in brackets. Superimposition of the ten chromatograms (B) shows a concentration dependent shift of the peak to higher molecular weight as sample concentration is increased. Values of estimated molecular weight and protein concentrations at the peak were fitted to a saturation-binding curve (C). The molecular weight estimated at saturation is consistent with the molecular weight of the heterotetramer (93 KDa). Values of the molecular weight for the monomeric proteins VARP (21.5 KDa) and Rab32 (25 KDa), heterodimer (46.5 KDa) and heterotetramer (93 KDa) are indicated.

(D) IF confocal microscopy of VARP-GFP-HeLa cells without cytosol extraction (relating to Figure 2H) (GFP (green), VPS26 (red), nuclei (blue)) showing that disrupting the VAMP7 and/or Rab32 interaction with VARP did not inhibit the recuitment of VARP onto endosomes. Boxed regions in the merged panels are shown as separate green (top), red (middle) and merged (bottom) channels on the right. Scale bar = 20μm

Figure S3. VARP is recruited to endosomes by retromer. Related to Figure 3

A

VPS29-GFP
ESSEEPLAPSDPFSLK

GFP-VPS35
ESSEEPLAPSDPFSLK, QGSLSAKPPESEGFGDR,
MSLLSQMTSSPTDCLFK, LNDPSVVTPFSR,
AVADGDLEMVR, TAVDCAEQNSK,
HTVEDAVVSQGPEAAGPLSTPQEVSASR

B

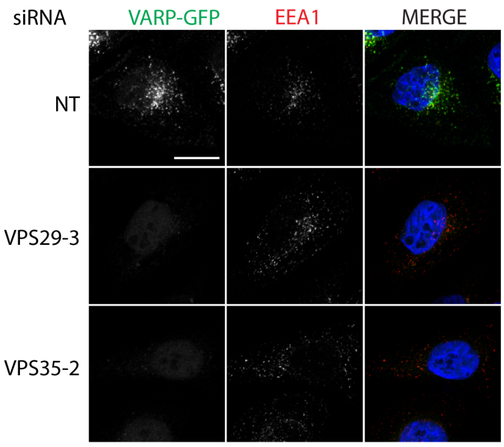

C

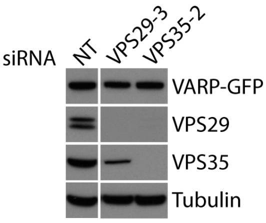

D

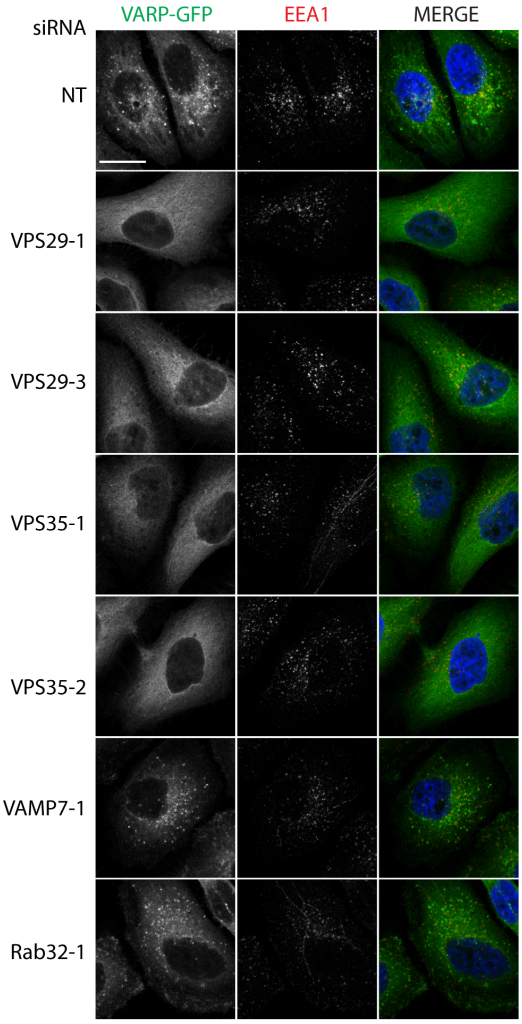

E

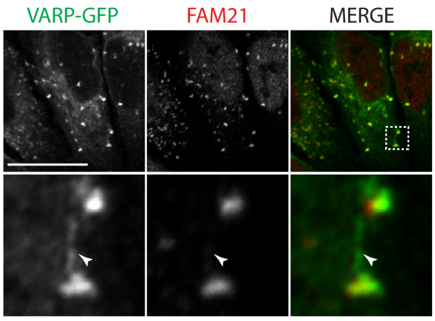

F

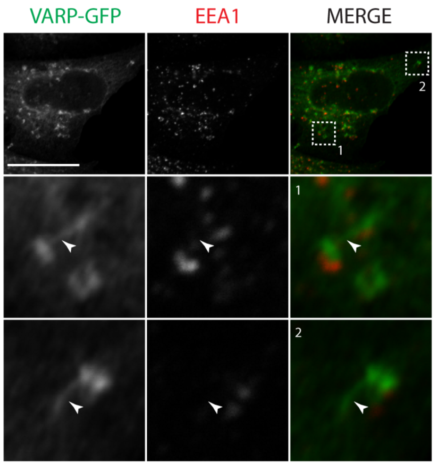

FIGURE S3

(A) VARP peptides identified by anti-GFP native co-immunoprecipitation and mass spectrometry analysis using HeLa cells stably expressing VPS29-GFP or GFP-VPS35. No VARP peptides were detected in a similar control lysate of untransfected HeLa cells treated with anti-GFP.
(B) IF confocal microscopy of cytosol extracted VARP-GFP-HeLa cells knocked-down with single siRNA oligonucleotides at 100nM (NT = non-targeting control, VPS29-3, VPS35-2), (GFP (green), EEA1 (red), nuclei (blue, merged panels)).
(C) Western blots of the cells imaged in B showing successful protein depletion. The VPS29-3 and VPS35-2 images are from cropped regions of the same Western blots that appear in Figure 3B, and the NT images are identical to those in Figure 3B.
(D) IF confocal microscopy of VARP-GFP-HeLa cells without cytosol extraction relating to Figure 3A and S3B (GFP (green), EEA1 (red), nuclei (blue, merged panels)).
(E)(F) IF confocal microscopy of VARP-GFP-HeLa cells without cytosol extraction (GFP (green), FAM21 (red)(E) or EEA1 (red)(F)). The boxed regions are shown in the bottom row with the VARP-GFP positive tubular domains of endosomes indicated with arrowheads. Scale bars = 20μm

Figure S4. VARP binds to a conserved hydrophobic patch on VPS29, which also binds to TBC1D5.
Related to Figure 4

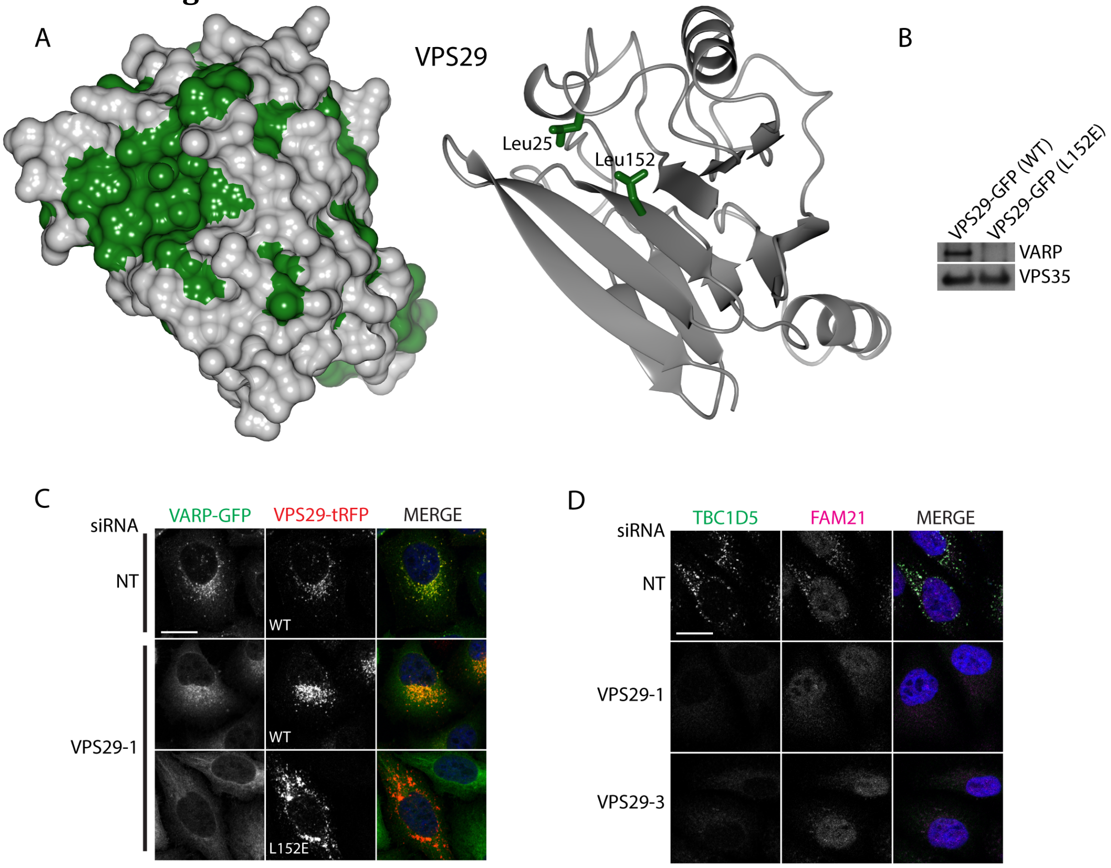

FIGURE S4

(A) Structure of VPS29 in surface representation (PDB code 1Z2W) with exposed hydrophobic side chains coloured in green (left panel) and in ribbon representation with Leu25 and Leu152, mutation of which abolishes both VARP and TBC1D5 binding, indicated (right panel).

(B) Anti-GFP native co-immunoprecipitation of HeLa cells expressing wild-type (WT) or L152E mutant VPS29-GFP, Western blotted for VARP and VPS35.

(C) IF confocal microscopy of VARP-GFP-HeLa cells without cytosol extraction relating to Figure 4D.

(D) IF confocal microscopy of cytosol extracted VARP-GFP-HeLa cells knocked-down using single siRNA oligonucleotides at 100nM (NT = non-targeting control, VPS29-1, VPS29-3) (TBC1D5 (green) and FAM21 (magenta), nuclei (blue, merged panels)). Scale bars = 20µm

Figure S5. VARP binds to VPS29 via two conserved Zn^{2+} coordinating Cys-rich motifs.
Related to Figure 5

A

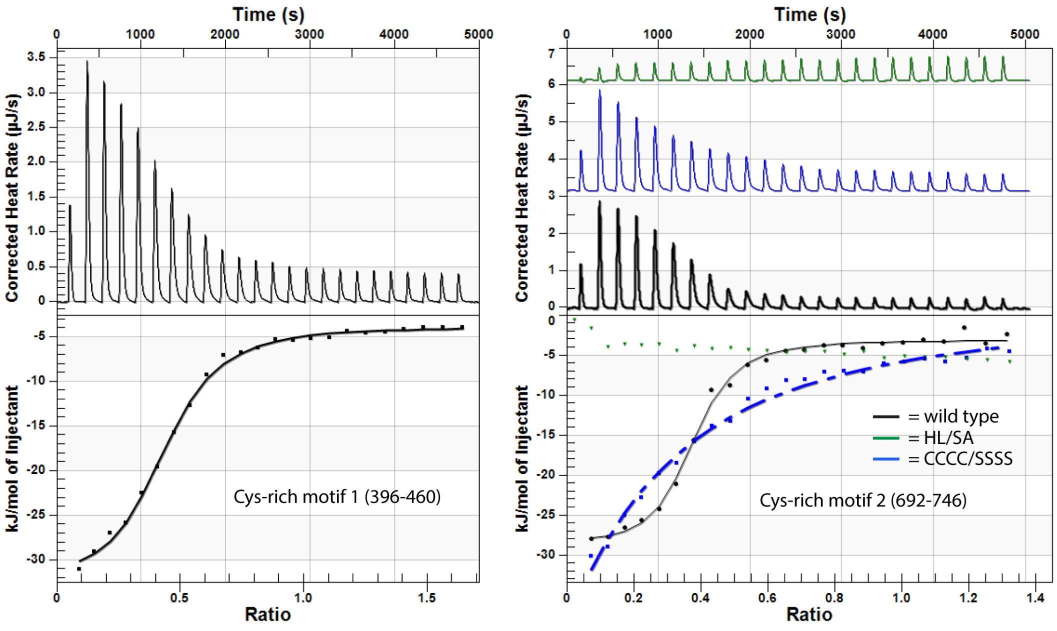

B

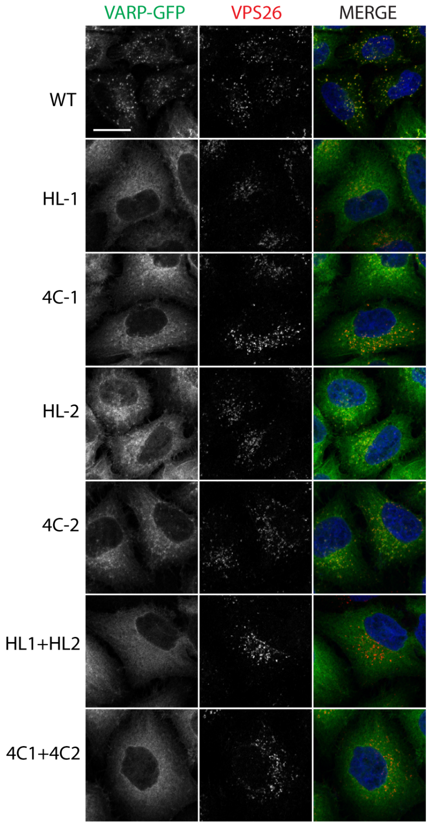

FIGURE S5

(A) Isothermal titration calorimetry (ITC) traces of VARP Cys-rich motif 1 (residues 396-460) and motif 2 (residues 692-746) binding to VPS29. For motif 2, H712S/L714A (HL/SA) (green trace) and C711S/C715S/C717S/C720S (CCCC/SSSS) (blue trace) mutations were tested.
(B) IF confocal microscopy of HeLa cells expressing wild type (WT) or mutated VARP-GFP, without cytosol extraction, related to Figure 5E. Scale bar = 20μm.

Figure S6. The VARP/retromer/VAMP7 protein network is required for GLUT1 trafficking.
Related to Figure 6

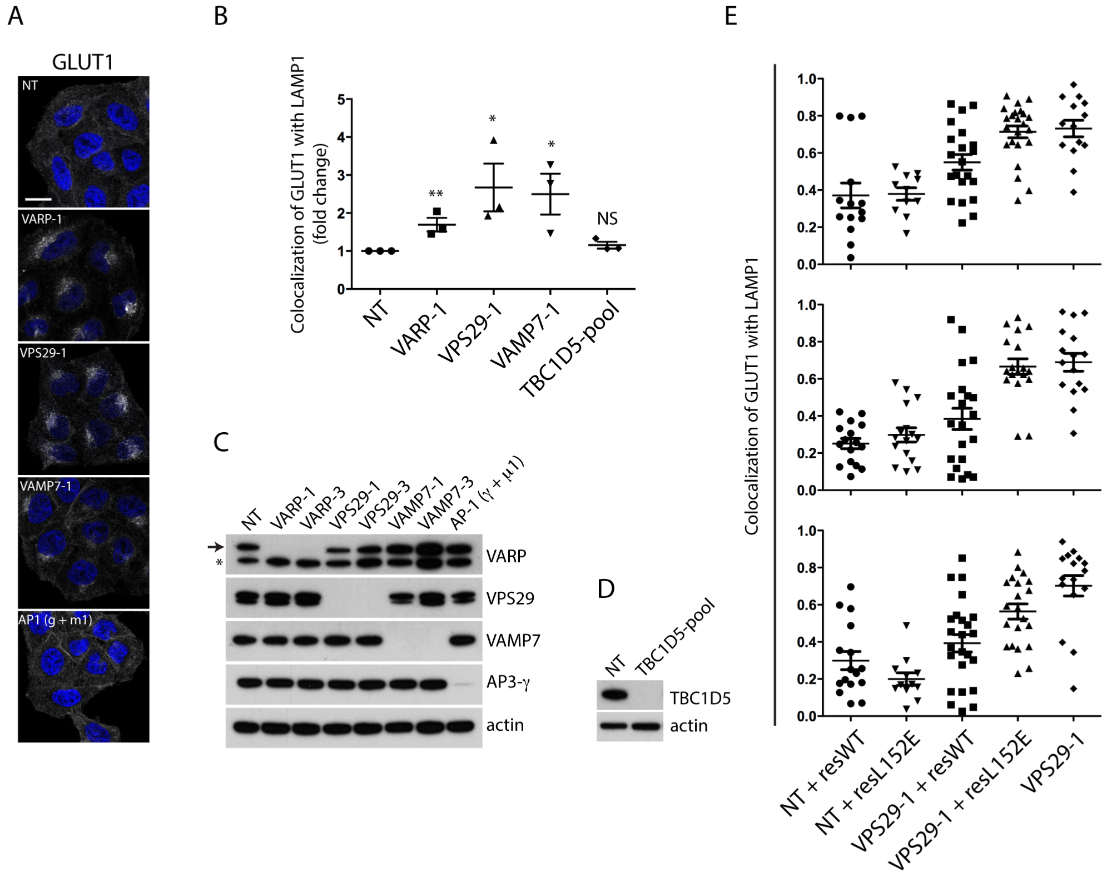

FIGURE S6

(A) Representative IF confocal microscopy of cells imaged in Figure 6A (GLUT1 (white), nuclei (blue)). Scale bar = 20μm.

(B) Fold-changes of colocalization coefficient measuring the colocalization of GLUT1 with LAMP1 after knock-down of VARP, VPS29, VAMP7 and TBC1D5. Mean (+/- SEM) of 3 experiments as described in Figure 6B and 6C (* = $p \leq 0.05$, **=$p<0.02$, NS = not significant).

(C and D) Western blots confirming successful protein depletion after knock-down with siRNA oligonucleotides as indicated. * = non-specific band.

(E) Individual data points and means (+/- SEM) of LAMP1 vs GLUT1 colocalisation coefficients for the 3 individual experiments used for Figure 6D.

Table S1 Crystallographic data for VARP ANKRD1:Rab32SeMet(Q85L) and VARP ANKRD1:SeMetRab38(Q69L) crystals
Related to Table 1

	VARP:SeMet Rab32	VARP:SeMet Rab38
Space group	C2	C2
No. complexes in ASU	3	6
Unit cell (Å)	*a*=113.0 *b*=122.5, *c*=131.6	*a*=258.2, *b*=83.9, *c*=161.2
(˚)	β=113.25	β=122.42
Wavelength (Å)	0.9173	0.9795
Resolution range (Å)	57.05-2.97 (3.12-2.97)	80.19-4.50 (5.03-4.50)
Beamline	IO4-1	I03
Number of crystals	1	1
R_{merge}	0.385 (2.096)	0.370 (1.373)
R_{merge} in top intensity bin	0.061	0.049
R_{meas}	0.420 (2.279)	0.414(1.727)
R_{pim}	0.163 (0.883)	0.182 (0.756)
Number of total reflections	211443 (28839)	88684 (25413)
Number of unique reflections	33107 (4412)	17604 (4941)
Mean ([I]/sd[I])	7.7 (1.9)	4.7 (1.4)
Half-dataset correlation coefficient $CC_{1/2}$	0.964 (0.395)	0.978 (0.539)
Completeness (%)	97.4 (98.4)	99.8 (99.6)
Anomalous completeness (%)	96.6 (97.5)	99.2 (99.0)
Multiplicity	6.4 (6.5)	5.0 (5.1)
Anomalous multiplicity	3.2 (3.3)	2.6 (2.6)
D_{anom} Correlation between half-sets	-0.222 (inner 0.332)	0.046 (inner 0.508)
Wilson plot B (Å^2) [a]	38.4	108.5

$R_{merge} = \Sigma(I_{hl} - <I_h>)/\Sigma <I_h>$

$R_{meas} = \Sigma\sqrt{}(n_h/n_h-1)(I_{hl} - <I_h>)/\Sigma <I_h>$

$R_{pim} = \Sigma\sqrt{}(1/n_h-1)(I_{hl} - <I_h>)/\Sigma <I_h>$

[a] “Inner” resolution range 80.18 to 10.06 Å

Table S2 Proton Induced X-ray Emission data
Related to Figure 5

		Ca	Fe	Cu	Zn
ANKRD1	Metal atoms/ monomer	0.021±0.003	0.014±0.005	0.008±0.005	3.0±0.2
	av/MDL	4.2	6.2	1.2	296
ANKRD2	Metal atoms/ monomer	0.011±0.002	0.003±0.002	0.007±0.004	1.12±0.08
	av/MDL	1.9	1.2	1.7	95
Buffer	av/MDL	1.1	2.9	10.9	2.5

MicroPIXE results. The stoichiometry is calculated as an average of 4 individual measurements (Av). Errors on the number of atoms/protein monomer are 1 standard deviation from this average. Av/MDL (minimum detectable limit) indicates the significance of the result: <1, not present, 1-3, potentially present, >3, present at a methodologically significant level. The levels in the buffer indicate that the only detectable impurity (~ppm level) is Cu.

Supplementary Materials and Methods

DNA Constructs for crystallization and protein:protein interaction studies

All genes used in this study are human with the exception of Rab38 and retromer subunits, which were murine. Constructs used in this study: pMWVARP$_{1-1050}$His$_{10}$ WT and mutants, pGEX-6P1VARP$_{397-650}$His$_{6}$, pGEX-6P1VARP$_{421-650}$His$_{6}$, pGEX-6P1VARP$_{451-640}$His$_{6}$, WT and mutants, Q509A, L513D, K546D, Y550A, Q509A/Y550A, L513D/K546D, pGEX-6P1Rab38$_{1-188}$(Q69L mutant), pGEX-6P1Rab32$_{1-225}$ WT and mutants Q85L, T39N, Q85L/M91S/R91S, Q85L/G89T/N90S/V94L, Q85L/V100M/Q153M/V158M/I192M, pGEX-6P1Rab5$_{1-215}$ (Q79L mutant), pMWRab7$_{1-204}$(Q67L mutant), pGEXRab7L1$_{1-203}$(Q67L). pGEX4T2VPS29, pGEX4T2VPS35+Vps29, pMWHis$_{6}$VPS26, pGEX4T2VPS35+His$_{6}$VPS26, pGEX6P1 VARP 396-460, pGEX6P1 VARP 692-746 pGBT9 VPS26A, pGBT9 VPS26B, pGBT9VPS29 (WT, L152E, I91S), pGBT9 VPS35, pGAD-CVARP (1-1050, 1-135, 1-410, 1-450, 369-450, 400-450, 410-450, 692-730, 1-1050(HL1), 1-1050(HL2), 1-1050(HL1+HL2), 410-450(HL1), 692-730(HL2), 410-450(4C1), 692-730(4C2))

DNA constructs used for cell biology

pEGFP-N3 VARP-EGFP and pLXIN VARP-EGFP (WT, Q509A/Y550A(QY), L513D/K546D(LK), M684D/Y687S(MY), Q509A/Y550A/M684D/Y687S(QY+MY), C431S/C435S/C437S/C440S(4C1), C711S/C715S/C717S/C720S(4C2), C431S/C435S/C437S/C440S/C711S/C715S/C717S/C720S(4C1+4C2), H432S/L434A(HL1), H712S/L714A(HL2), H432S/L434A/H712S/L714A(HL1+HL2))

pTagRFP-N VPS29-TagRFP (WT, L152E), pIRESneo2 VPS29-EGFP (WT, L152E), pIRESneo2 EGFP-VPS35, pLXIN VAMP7-2xHA (WT, D69A/E71F/S73D)

Full crystallization and structure determination

Crystals of $VARP_{451-640}His_6$: $Rab32_{1-225}$(Q85L) and of VARP-ANKR domain 1:SeMet- $Rab32_{1-225}$(Q85L/V100M/Q153M/V158M/I192M) were grown by sitting drop vapor diffusion at 18°C against well solutions containing 14-19% (w/v) of PEG 3350, 200 mM sodium citrate pH 8.0 at an initial complex concentration of 2.5mg/ml. Xe-derivatized crystals were produced by placing crystals in a sealed chamber into which xenon was forced to 10–15 atmospheres pressure for 1-2 min. Small crystals of $VARP_{451-640}His_6$:SeMet-$Rab38_{1-188}$(Q69L) complex were grown by sitting drop vapor diffusion at 18°C from complex at a concentration of 3.8 mg/ml against a reservoir containing 16% (w/v) of PEG 3350 and 200 mM sodium citrate pH 7.0. Larger crystals were grown by streak seeding.

All crystals were cryoprotected in their mother liquor containing 25 % (v/v) glycerol and flash cooled in liquid nitrogen. Diffraction data were collected at 100 K at Diamond Light Source plc. Crystals exhibited anisotropic diffraction and suffered severe radiation damage. Complete data sets often required collection of several data sets from different regions of the crystals Tables S1 and S2.

Datasets were indexed and integrated with Mosflm (Leslie, 2006), and scaled and reduced with Aimless (Evans, 2011). VARP-ANKR domain 1:Rab32 (Q85L) crystals belonged to the space group $P3_221$ and diffracted in the best directions to 2.8Å. The structure was solved by the Molecular Replacement with

Phaser (McCoy et al., 2007) using models based on the structures of Rab7A (PDB 1YHN) and the VARP ANKRD2 (PDB 4B93). A single solution was obtained with 3 molecules of complex per asymmetric unit with electron density maps exhibiting good connectivity for the three complexes and clear density for GppCp:Mg^{++}. Xe sites were calculated using Phaser and the anomalous phases used as restraints in initial rounds of refinement.

Crystals of VARP-ANKR domain 1:SeMet-Rab32 (Q85L) and of VARP-ANKR domain 1:SeMet-Rab38 (Q69L) both belonged to the space group C2, (see Table 1) and diffracted in their best directions to 3Å and 4.5Å respectively. Their structures were solved by Molecular Replacement using the coordinates of native VARP ANKRD1:Rab32(Q85L) complex as the search model. VARP ANKRD1:SeMet-Rab32(Q85L) consisted of three molecules of complex per asymmetric unit, whilst that of ANKR domain 1:SeMet-Rab38(Q69L) consisted of six molecules of complex per asymmetric unit. No further refinement of ANKR domain 1:SeMet-Rab38(Q69L) structure was undertaken due to the low resolution of the data.

Model building and refinement were carried out with Coot (Emsley et al., 2010) and Refmac (Murshudov et al., 2011) and the geometry of final models checked using MolProbity (Chen et al., 2010). Analysis of protein interfaces was performed with PISA (Krissinel and Henrick, 2007). Structure pictures were prepared with *PyMOL* [HTTP://WWW.PYMOL.ORG/].

Antibodies

GFP (IF: mAb and pAb, Molecular Probes, IP: anti-GFP pAb (Seaman et al., 2009)), VARP (pAb (Schafer et al., 2012)), VPS29 (goat pAb, Abcam), Rab32 (rabbit pAb,

gift from Miguel Seabra), VPS35 (mAb B-5, Santa Cruz), VAMP7 (IF: anti longin domain pAb (Pryor et al., 2008)), IB: mAb, gift from Andrew Peden), TBC1D5 (mAb E-9, Santa Cruz), FAM21 (pAb S-13, Santa Cruz), Tubulin (mAb DM1A, Sigma), Actin (rabbit pAb, Sigma), VPS26 (pAb (Seaman, 2004)), AP-1 γ-subunit (mAb 100.3, gift from Margaret S. Robinson), GLUT1 (pAb 15309, Abcam).

siRNA mediated gene knock-down

siRNA oligonucleotides targeting human genes were designed and synthesized by Dharmacon/Thermo Scientific. Cells were transfected (two transfections separated by 48hrs) using Oligofectamine (Life Technologies) according to manufacturer's instructions. On-Target Plus oligonucleotide catalogue numbers were as follows: non-targeting (NT) control (D-001810-01), VARP-1 (J-014788-09), VARP-3 (J-014788-11), VPS29-1 (J-009764-09), VPS29-3 (J-009764-11), VPS35-1 (J-010894-05), VPS35-2 (J-010894-06), VAMP7-1 (J-020864-05), VAMP7-3 (J-020864-07), Rab32-1 (J-009920-06), Rab32-2 (J-009920-07), TBC1D5 SMARTpool (L-020775-01), AP-1-γ SMARTpool (L-019183-00), AP-1-μ1 SMARTpool (L-013196-00).

Analytical Gel Filtration

Samples of reconstituted VARP-ANKR domain 1:Rab32 (Q85L) complex were run on a Superdex 75 PC3.2/30 using an AKTA Micro chromatography system. 50 μl protein samples at ten different concentrations varying from 0.1 μM to 150 μM were injected onto the column. Elution profiles were followed by measuring absorbance at 280nm and 230nm. The values of estimated molecular

weight and protein concentration in the peak were fitted to a saturation-binding curve using the program Prism.

Proton-induced X-ray emission

Proton-induced X-ray emission (PIXE) is an analytical tool for the unambiguous identification of atoms in a sample from the unique energy of their fluorescent X-rays, emitted on bombardment of the sample by energetic protons. These X-rays are detected in a lithium-drifted silicon detector with high-energy resolution. Stoichiometric quantitation is achieved for proteins by taking the ratio of the counts in the X-ray spectrum due to sulphur (originating from the known number of cysteines and methionines in the sequence) to the counts of the element of interest, in this case zinc (Garman and Grime, 2005; Garman and Zeldin, 2013).

Samples for analysis were gel filtered at 10 mg/ml into 20mM Tris pH 7.4, 100mM NaBr and 0.5mM TCEP in order to ensure low chlorine content and no non-protein sulphur. The low chlorine content is necessary because if the chlorine peak is large, its tail overlaps with the neighbouring peak from sulphur. This has the effect of increasing the lower limit of detection for sulphur. 0.2 µl of each sample was placed onto a 4 µm thick prolene film, and left to dry overnight. For the microPIXE analysis, a 2.5 MeV proton beam of diameter 2 µm was used and was scanned across the drop in the x and y directions. The resulting X-ray spectra were sorted into 2-D elemental maps. Both sulphur and zinc were clearly detectable and allowed the protein to be located for the selection of 4 suitable points for measurements from the sulphur (protein) rim of the map. The resulting spectra were analyzed in OMDAQ (Grime and Dawson, 1994). Sulphur-

normalisation was performed for all metals with potentially significant values, and the means and standard deviations for the four point measurements on each sample were calculated. Metal signals were deemed significant if the ratio of peak counts to minimum detectable limit (MDL), as determined by GUPIX (Maxwell et al., 1989), was >3, and of interest, but not significant, if between 1 and 3. The results are shown in Table S2.

Supplementary References

Chen, V.B., Arendall, W.B., 3rd, Headd, J.J., Keedy, D.A., Immormino, R.M., Kapral, G.J., Murray, L.W., Richardson, J.S., and Richardson, D.C. (2010). MolProbity: all-atom structure validation for macromolecular crystallography. Acta Crystallogr D Biol Crystallogr *66*, 12-21.

Emsley, P., Lohkamp, B., Scott, W.G., and Cowtan, K. (2010). Features and development of Coot. Acta Crystallogr D Biol Crystallogr *66*, 486-501.

Evans, P.R. (2011). An introduction to data reduction: space-group determination, scaling and intensity statistics. Acta Crystallogr D Biol Crystallogr *67*, 282-292.

Garman, E.F., and Grime, G.W. (2005). Elemental analysis of proteins by microPIXE. Progress in biophysics and molecular biology *89*, 173-205.

Garman, E.F., and Zeldin, O.B. (2013). Elemental Analysis of Proteins by Proton Induced X-ray Emission (microPIXE). Advancing Methods for Biomolecular Crystallography 79–89.

Grime, G.W., and Dawson, M. (1994). A PC-based data acquisition package for nuclear microbeam systems. Nuclear Instruments and Methods in Physics Research Section B *89*, 223-228.

Krissinel, E., and Henrick, K. (2007). Inference of macromolecular assemblies from crystalline state. J Mol Biol *372*, 774-797.

Leslie, A.G. (2006). The integration of macromolecular diffraction data. Acta Crystallogr D Biol Crystallogr *62*, 48-57.

Maxwell, J., Campbell, J., and Teesdale, W. (1989). The Guelph PIXE software package. Nuclear Instruments and Methods in Physics Research Section B *43*, 218-230.

McCoy, A.J., Grosse-Kunstleve, R.W., Adams, P.D., Winn, M.D., Storoni, L.C., and Read, R.J. (2007). Phaser crystallographic software. J Appl Crystallogr *40*, 658-674.

Murshudov, G.N., Skubak, P., Lebedev, A.A., Pannu, N.S., Steiner, R.A., Nicholls, R.A., Winn, M.D., Long, F., and Vagin, A.A. (2011). REFMAC5 for the refinement of macromolecular crystal structures. Acta Crystallogr D Biol Crystallogr *67*, 355-367.

Pryor, P.R., Jackson, L., Gray, S.R., Edeling, M.A., Thompson, A., Sanderson, C.M., Evans, P.R., Owen, D.J., and Luzio, J.P. (2008). Molecular basis for the sorting of the SNARE VAMP7 into endocytic clathrin-coated vesicles by the ArfGAP Hrb. Cell *134*, 817-827.

Schafer, I.B., Hesketh, G.G., Bright, N.A., Gray, S.R., Pryor, P.R., Evans, P.R., Luzio, J.P., and Owen, D.J. (2012). The binding of Varp to VAMP7 traps VAMP7 in a closed, fusogenically inactive conformation. Nat Struct Mol Biol *19*, 1300-1309.

Seaman, M.N. (2004). Cargo-selective endosomal sorting for retrieval to the Golgi requires retromer. J Cell Biol *165*, 111-122.

Seaman, M.N., Harbour, M.E., Tattersall, D., Read, E., and Bright, N. (2009). Membrane recruitment of the cargo-selective retromer subcomplex is catalysed by the small GTPase Rab7 and inhibited by the Rab-GAP TBC1D5. J Cell Sci *122*, 2371-2382.
